# Predicting therapeutic responses in metastatic colorectal cancer through personalized functional profiling of patient-derived spheroids

**DOI:** 10.1038/s41698-026-01356-7

**Published:** 2026-03-19

**Authors:** Victoria El-Khoury, Lejla-Nur Smajović, Takouhie Mgrditchian, Vanessa Barthelemy, Pauline Torigny, Hichul Kim, Jean-Jacques Gerardy, Petr V. Nazarov, Jérôme Graas, Myriam Menster, Katrin B. M. Frauenknecht, Bob Edon, Boris Meuter, Bernard Faber, Jan Friedrich Krahn, Samer Abi-Khalil, Barbara Klink, Daniel Stieber, Felix Bruno Kleine Borgmann, Guy Berchem, Michel Mittelbronn, Marc Berna, Yong-Jun Kwon

**Affiliations:** 1 Precision Medicine Technology, Translational Medicine Operations Hub, Luxembourg Institute of Health, Dudelange, Luxembourg; 2 National Center of Genetics, Laboratoire National de Santé, Dudelange, Luxembourg; 3 Department of Cancer Research, Cytoskeleton and Cancer Progression, Luxembourg Institute of Health, Luxembourg, Luxembourg; 4 National Center of Pathology, Laboratoire National de Santé, Dudelange, Luxembourg; 5 Bioinformatics and AI Unit, Department of Medical Informatics, Luxembourg Institute of Health, Strassen, Luxembourg; 6 Multiomics Data Science, Department of Cancer Research, Luxembourg Institute of Health, Strassen, Luxembourg; 7 Clinical and Epidemiological Investigation Center, Translational Medicine Operations Hub, Luxembourg Institute of Health, Strassen, Luxembourg; 8 Luxembourg Center of Neuropathology, Luxembourg Institute of Health, Dudelange, Luxembourg; 9 Institute for Neuropathology, University Medical Center of the Johannes Gutenberg University Mainz, Mainz, Germany; 10 Hôpitaux Robert Schuman, Luxembourg, Luxembourg; 11 Fondation Hôpitaux Robert Schuman, Luxembourg, Luxembourg; 12 Department of Radiology, University hospital of Angers, Angers, France; 13 Univ Angers, Inserm, CNRS, MITOVASC, Equipe CarMe, SFR, ICAT, Angers, France; 14 Luxgen Genome Center, Translational Medicine Operations Hub, Luxembourg Institute of Health, Dudelange, Luxembourg; 15 Department of Life Sciences and Medicine, Faculty of Science, Technology and Medicine, University of Luxembourg, Esch-sur-Alzette, Luxembourg; 16 Saarland University Medical Center, Homburg, Germany; 17 Luxembourg Institute of Health, Strassen, Luxembourg; 18 Centre Hospitalier de Luxembourg, Luxembourg, Luxembourg

**Keywords:** Cancer, Computational biology and bioinformatics, Drug discovery, Oncology

## Abstract

Drug resistance of metastatic colorectal cancer (mCRC) remains a major therapeutic challenge. Screening patient-derived tumor cells with diverse compounds in 3D models may overcome the limitations of genomics-based drug response predictions. We describe a personalized functional profiling (PFP) approach in mCRC using patient-derived spheroids (PDS) and assess its utility in predicting drug responses. PDS were established from twelve patients’ tumors and validated by immunohisto(cyto)chemistry and genomic sequencing. Forty-two small molecule anti-cancer drugs, along with five standard-of-care (SOC) drugs in CRC were screened as single agents or in combination, and cell viability was measured using calcein staining or ATP-based assay. Ex vivo results were compared with clinical treatment responses. PDS closely mirrored histopathological and genetic features of the original tumors, supporting their use in PFP. Sensitivity to anti-EGFR drugs distinguished responsive from resistant patients and revealed candidates for anti-ERBB2 therapy, whereas anti-VEGFR screening failed to recapitulate clinical outcomes. SOC drug screening results correlated with clinical outcomes or tumor genetic features in a subset of PDS. This work underscores the predictive value of PFP, its complementarity with genomic sequencing, and the need for refinement to enhance its clinical applicability.

## Introduction

Colorectal cancer (CRC) is the third most common and the second deadliest cancer worldwide^1^. Approximately 1.93 million new cases were diagnosed (9.6% of global cancer incidence) and 0.94 million CRC-related deaths (9.3% of all cancer deaths) recorded in 2022^2^. Whereas incidence and mortality of CRC have declined in individuals over 50, probably due to the implementation of screening programs, the significant increase in cases among younger adults is alarming^1^. Advances in early diagnosis and treatment options have improved the survival of patients with CRC^3^. Yet, the 5-year survival of patients with metastatic CRC (mCRC) for whom no local therapy is available falls below 15%^3,4^.

In addition to immunotherapy, the first-line treatments of mCRC include both traditional chemotherapeutic agents, mostly 5-fluorouracil-containing regimens, and targeted agents, namely bevacizumab (anti-VEGF-A antibody), cetuximab or panitumumab (anti-EGFR antibodies). Subsequent therapy options include agents not administered in first line such as encorafenib in BRAF V600E-mutated tumors, ramucirumab or aflibercept (antiangiogenic agents), TAS-102 (trifluridine-tipiracil) and the multikinase inhibitor regorafenib^5^. The resistance to currently available anticancer agents remains a major treatment challenge. Therefore, the development of effective therapeutic approaches to fight mCRC represents an urgent medical need.

By transforming genomic data into actionable insights, DNA mutational analysis has significantly advanced personalized cancer medicine. Nevertheless, genomics alone is often insufficient to identify effective treatments for patients with advanced disease, as evidenced by the modest 13% of patients experiencing objective response to molecularly targeted drugs^6,7^. Moreover, in 30–60% of patient samples, genetic analysis does not reveal a druggable alteration, and only 9–23% of patients with advanced solid tumors ultimately receive matched targeted therapy, which represents a major barrier to treatment for those who have exhausted standard-of-care approaches^8–11^. Functional profiling, namely drug screening, can overcome the limitations of genomics-based drug response prediction if performed in physiologically relevant tumor models^12–23^.

In this manuscript, we describe our personalized functional profiling (PFP) approach in mCRC (pilot clinical study NCT03997617) and provide proof of concept that personalized drug screening in patient-derived spheroids can predict treatment response and complement genomic analysis in elucidating tumor behavior to anticancer therapy. Our results pave the way for integrating PFP into a personalized cancer management approach to improve mCRC treatment.

## Results

### Descriptive metrics of the drug screening process

Patient-derived mCRC spheroids were successfully generated from 12 of 19 patient samples (63%). Two patient-derived cultures were discarded due to bacterial contamination, while the remaining samples failed to propagate in culture. Established spheroids were either cryopreserved for later use or directly utilized in downstream assays. When fresh spheroids were used in drug screening, the median time to cell printing was 34 days (IQR 29–55.8). The median overall turnaround time from sample collection to release of drug screening results was 55.5 days (IQR 36.8–90.3). In two cases (ID10 and ID12), technical issues required repetition of the drug screen, thereby prolonging the turnaround time. Excluding these two cases, the median turnaround time dropped to 39 days (IQR 36.3–94.3) The descriptive metrics of the drug screening process for each fresh spheroid sample are provided in Supplementary Table 1.

### Patient-derived mCRC spheroids recapitulate immunohistopathological characteristics of the parental tumor

**Fig. 1:**
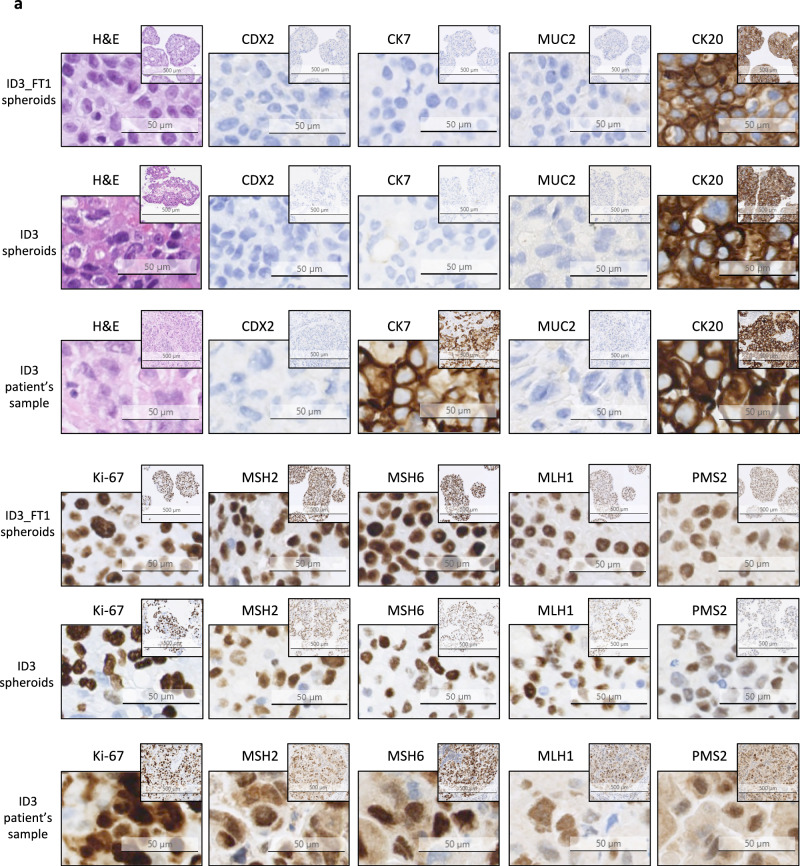

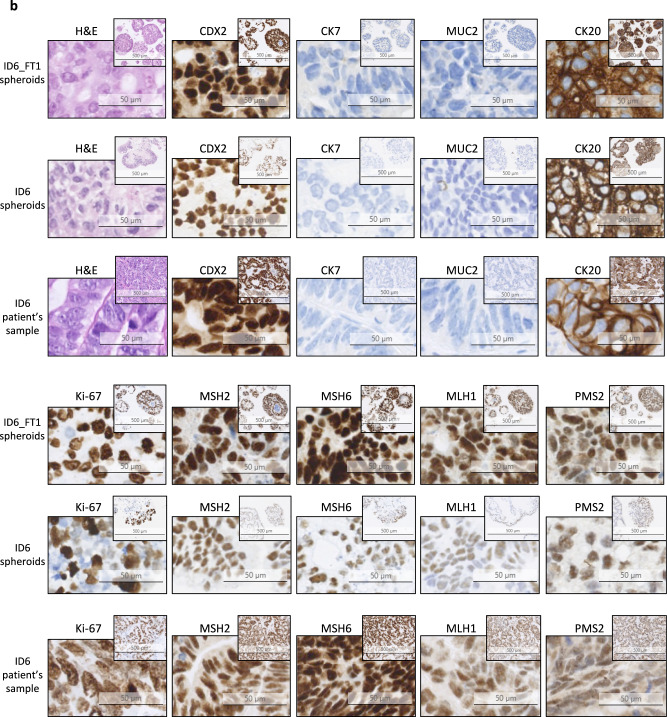
Fresh and cryopreserved patient-derived mCRC spheroids retain the histological features and immunophenotype of parental tumors. Representative images of hematoxylin-eosin staining (H&E) and immunohisto-/immunocytochemistry in 2 patient samples and their derived spheroids are shown. Stainings were performed on tissue or spheroid sections using antibodies against intestinal and/or epithelial antigens (CDX2, CK7, MUC2 and CK20), the proliferation marker Ki-67 and the mismatch repair proteins (MSH2, MSH6, MLH1 and PMS2). DAB was used for visualisation. A hematoxylin-eosin (H&E)-stained slide is presented for each sample. Images from ID3 (**a**) and ID6 (**b**) parental tumor and derived fresh and previously cryopreserved spheroids are shown. FT freeze-thaw cycle.

We first investigated whether both fresh and cryopreserved spheroids retain immunohistopathological characteristics of the parental tumor. Two representative sets of immunostaining results corresponding to patient samples ID3 and ID6 and their derived spheroids are shown in Fig. 1.

ID3 tumor sample was obtained from a mesenteric lymph node biopsy of a metastatic CRC. The hematoxylin and eosin (H&E) staining revealed notable morphological similarities between ID3 patient tumor and its matched fresh and cryopreserved spheroids, as shown by the presence of epithelial cells with large nuclei, prominent nucleoli and an increased nuclear-cytoplasmic ratio (Fig. 1a). The immunostaining analysis showed that the intestinal differentiation marker CDX2 and the mucin 2 marker MUC2 were expressed neither in ID3_FT1 nor in ID3 spheroids, whereas both samples were positive for cytokeratin 20 (CK20), an epithelial marker commonly expressed in colon adenocarcinoma. Similar staining profiles were observed in the corresponding patient’s tumor. In addition, ID3 patient’s cancer cells were highly proliferative, as shown by their extensive positivity to Ki-67, a feature maintained in the fresh and cryopreserved spheroids. The epithelial marker CK7 was not expressed in the spheroids, whereas the parental tumor was strongly CK7-positive. Importantly, previous immunohistochemical analyses performed in the clinic revealed that the primary ID3 tumor and its metastases in the central nervous system and in the liver were CK7-negative (data available upon request), suggesting the presence of tumor cells with heterogeneous CK7 expression rather than the loss of CK7 in culture. The mismatch repair proteins MSH2, MSH6, MLH1 and PMS2 were expressed in both ID3_FT1 and ID3 spheroids, in accordance with the results obtained in the patient biopsy.

ID6 tumor sample was collected from a liver metastasis of a CRC. Figure 1b shows immunostaining results of ID6 patient tumor and its corresponding fresh and cryopreserved spheroids. All the immunochemistry markers used showed that the patient tumor’s protein expression pattern was maintained in fresh and in cryopreserved spheroids, as was gross cell morphology in H&E staining.

These results suggest that patient-derived spheroids recapitulate key immunohistopathological features and protein expression patterns of the parental tumor and that cryopreservation of the spheroids does not alter these characteristics.

### Patient-derived mCRC spheroids capture the genetic heterogeneity of the parental tumor

**Fig. 2:**
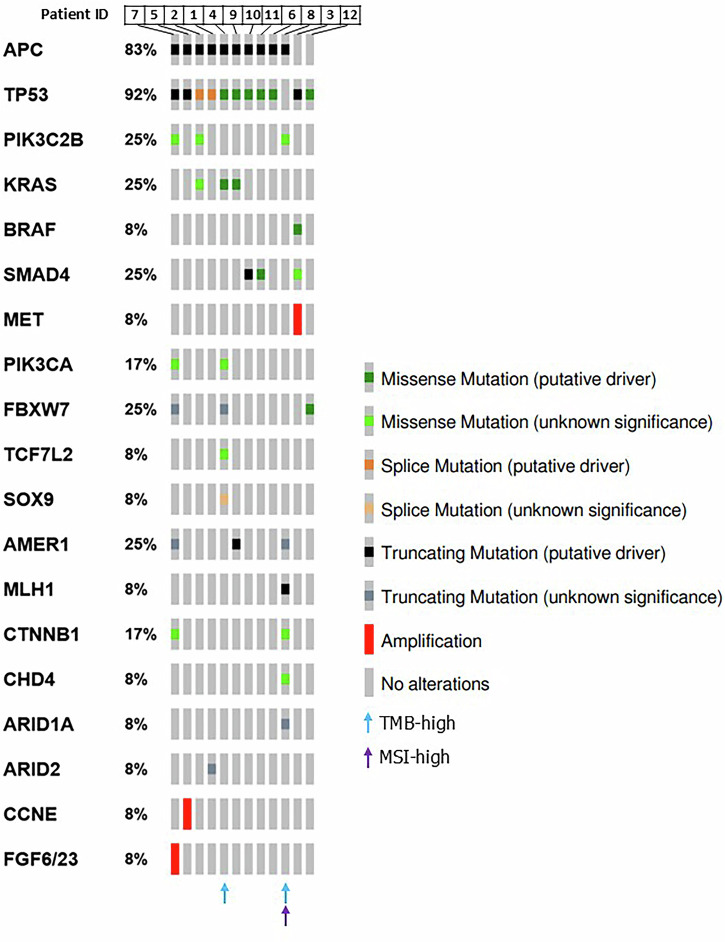
Oncoplot of key genetic alterations in the colorectal cancer cohort. The oncoplot shows recurrent genetic alterations across metastatic colorectal cancer samples, including putative driver mutations, copy number variations, and other frequent mutations. Each row represents a gene and each column represents a parental tumor sample. TMB-high and/or MSI-high statuses are indicated by arrows of different colors.

We analyzed the genomic landscape of twelve mCRC patient tumor samples and six matched spheroid samples. To characterize the parental tumors and identify potential therapeutic targets, tumor samples were analyzed using the TruSight Oncology 500 High-Throughput (TSO500 HT) kit for pathogenic variants across 523 genes of oncological significance. This analysis included assessment of microsatellite instability (MSI) status and tumor mutational burden (TMB). The tumors in this cohort exhibited characteristic genetic alterations commonly associated with CRC, with the analysis revealing several recurrently altered genes. Ten of twelve (83%) tumors exhibited mutations in *APC*, and 92% in the *TP53* gene (Fig. 2). Additionally, hot-spot mutations in *KRAS* were identified in 3/12 (25%) of tumors. Other noteworthy findings included loss-of-function mutations in *AMER1* identified in 3 of 12 tumors and recently associated with distant metastasis in colorectal cancer^24^. One tumor (ID8) was classified as microsatellite instability-high (MSI-high) and exhibited a high tumor mutational burden (TMB) of 65.0 Mut/Mb, along with a pathogenic truncating mutation in *MLH1*, confirming mismatch repair (MMR) deficiency in this case. Another tumor (ID4) also demonstrated a high TMB of 21.2 Mut/Mb; however, its microsatellite status remained stable (Fig. 2).

**Fig. 3:**
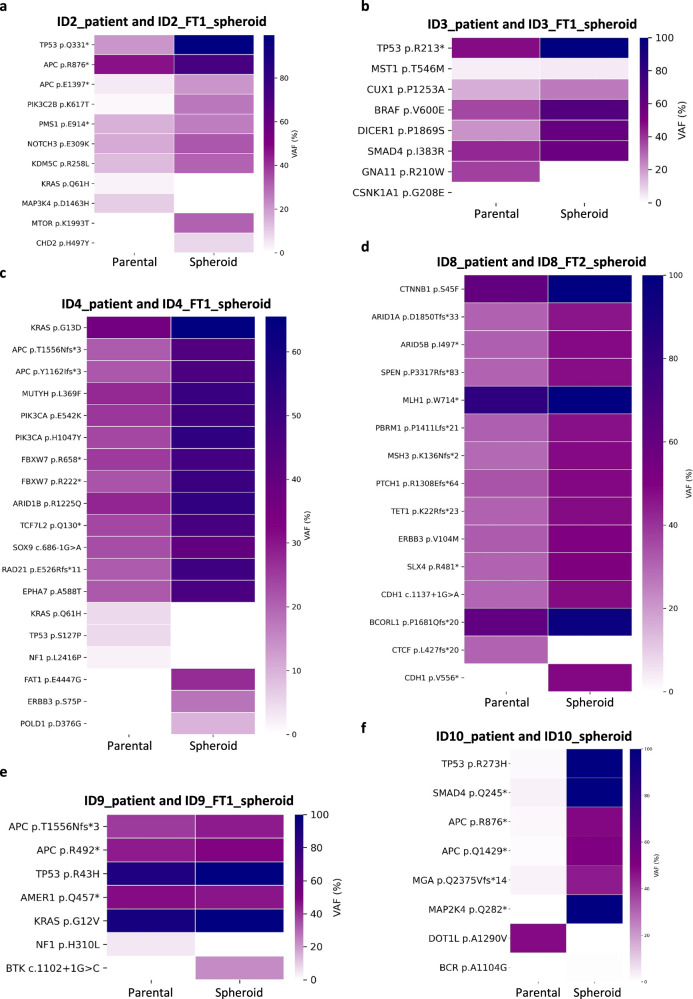
Comparison of variant allele frequencies (VAFs) in parental tumors and derived spheroids across all sample pairs. The heatmaps display the comparison of variant allele frequencies (VAFs) for shared and unique mutations across the six tumor and spheroid pairs analyzed in our cohort (**a**–**f**). Parental tumor is shown in the left column and matched spheroids in the right column of each pair.

The genomic analysis comparing original tumor samples with their corresponding spheroids revealed a high degree of genetic fidelity. A detailed list of shared and subclonal variants, including allele frequencies and sequencing depth, is provided in Supplementary Data 1. In the majority of cases, shared mutations exhibited higher variant allele frequencies (VAFs) in spheroids than in primary tumor samples, due to the high purity of tumor cells within the spheroid cultures as opposed to the heterogeneous nature of the patient samples containing normal tissue contamination (Fig. 3). Key driver mutations present in the original tumors, such as somatic alterations in *TP53*, *APC*, *KRAS*, were consistently detected in the derived spheroids across all sample pairs (Fig. 3). This congruence extended to structural variations, such as the *MET* amplification observed in sample ID3 and its matched spheroids (Supplementary Data 2). Copy number variation (CNV) analyses further confirmed this strong genomic alignment (Supplementary Fig. 1). Supplementary Table 2 provides an overview of shared and unique characteristics between tumors and corresponding spheroids. These findings underscore the spheroids’ value in preserving the genomic features of primary tumors, including both small variants and broader structural alterations.

Importantly, subclonal events with lower allele frequencies were observed in both tumor samples and spheroids. While some subclonal mutations were shared, such as APC p.E1397* mutation in ID2 pair (Fig. 3a) and MST1 p.T546M mutation in ID3 pair (Fig. 3b), others differed between the two, reflecting the inherent genetic heterogeneity of tumors (Fig. 3 and Supplementary Data 1). This partial overlap in subclonal variants indicates that spheroids maintain the complexity of subclonal heterogeneity seen in the original tumors, though some differences are expected given the inherent spatial heterogeneity of tumors. While selective pressures in culture may also play a role, the short duration of spheroid culture emphasizes that these differences primarily reflect the original tumor’s genetic heterogeneity. For instance, low-frequency *KRAS* missense driver mutations detected in primary tumors ID2 and ID4, with allele frequencies of 2.6% and 5%, respectively, were absent in the corresponding spheroids, whereas a novel *MLLT3::C6orf222* fusion was identified in the ID9_FT1 spheroids but not in its tumor counterpart (Supplementary Data 2).

Notably, Fig. 3f presents the mutational profile of ID10, for which both the patient tumor sample and derived spheroids were sequenced. Here, the VAFs in the patient sample were exceptionally low, suggesting limited tumor content in the sequenced material. In contrast, the matched spheroid sample displayed significantly higher VAFs for multiple key pathogenic alterations, underscoring the utility of spheroid models in amplifying tumor-specific signals that may otherwise be underrepresented or undetectable in low-purity samples.

### Fresh and cryopreserved patient-derived mCRC spheroids yield comparable drug screen results

It has been reported that cryopreservation and thawing can affect not only cell viability but also the cellular composition of a sample, cell functionality and phenotype, whereas other studies have shown that multiple rounds of cryopreservation do not alter cell function^25,26^. In order to test whether cryopreserved spheroids generate similar functional profiles as their fresh counterparts, we compared the dose-effect results of 33 molecules included in the 42-drug library (Supplementary Table 3) on fresh spheroids ID9 and on ID9_FT1 spheroids that underwent a freeze-thaw cycle after one year of cryopreservation. As shown in Supplementary Fig. 2, the matched AUC values obtained from ID9 and ID9_FT1 after a 5-day incubation time with the drugs were strongly correlated (Pearson correlation coefficient *r* = 0.9645, 95% confidence interval (0.9287, 0.9825), *p* value < 0.0001).

These data suggest that fresh and cryopreserved patient-derived spheroids are both suitable for drug profiling studies. In the present work, we used the cells either fresh or after a maximum of two freeze-thaw cycles. Although additional freeze-thaw cycles may not necessarily impair cellular phenotype or function, this possibility was not evaluated, as it was beyond the scope of the present study.

### Personalized drug screening in mCRC patient-derived spheroids captures tumor-specific mutational characteristics

**Fig. 4:**
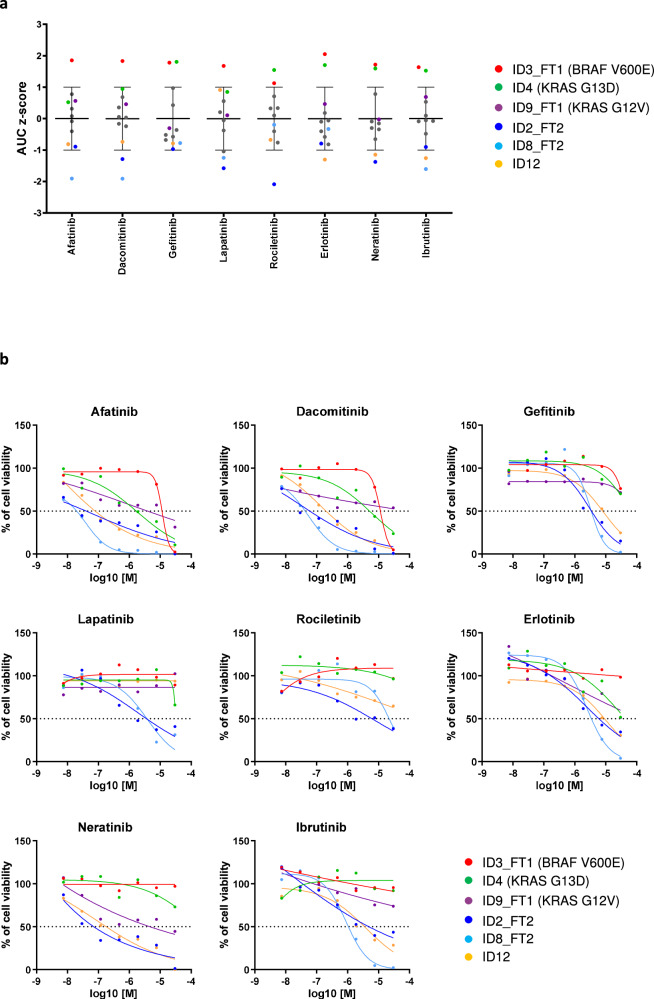
Personalized drug screening identifies mCRC patients whose tumors are genomically classified as resistant to anti-EGFR therapy and suggests potential responders to anti-EGFR or anti-ERBB2 therapy. The mCRC spheroids were subjected to drug screening as described in Methods. The data of the dose-response relationship generated with EGFR inhibitors and ibrutinib are presented. The scatterplot in (**a**) shows the individual AUC z-scores with their mean and standard deviation (*n* = 11–12) per drug. Each patient-derived spheroid sample is represented by a dot. For clarity, only six patient-derived spheroids are highlighted in the figure. In (**b**), the dose-response curves obtained with 6 patient-derived spheroids and 8 compounds are displayed. The graphs show the means (*n* = 2 to 4) and the fitted sigmoidal curve of each sample incubated with increasing concentrations of the indicated drugs. Data for rociletinib and neratinib were obtained from only 5 samples.

We investigated whether personalized functional profiling results of patient-derived spheroids are consistent with anti-EGFR drug responses based on the DNA mutational analysis. Of the 12 patients in the study cohort, three carried *KRAS* or *BRAF* mutations predictive of clinical resistance to anti-EGFR therapy: ID3 with BRAF p.Val600Glu (V600E), ID4 with KRAS p.Gly13Asp (G13D) and ID9 with KRAS p.Gly12Val (G12V) (Supplementary Data 1). The anti-EGFR drug response data of ID3_FT1, ID4 and ID9_FT1 spheroids showed high Area Under the Dose-Response Curve (AUC) z-scores and high absolute half-maximal inhibitory concentrations (IC_50_) compared to other patient samples, suggesting increased resistance, in accordance with the genomic analysis prediction (Fig. 4a, b). Interestingly, ID8_FT2 cells were highly sensitive to the dual EGFR/ERBB2 inhibitors afatinib, dacomitinib and lapatinib. The molecular analysis of this sample identified an ERBB3 p.V104M mutation (Supplementary Data 1) previously correlated with increased sensitivity to ERBB2 inhibition^27–29^. In addition, ID12 harbored an ERBB2 p.G776delinsVC mutation, which has been associated with sensitivity to irreversible ERBB2 inhibitors in lung cancer^30–32^. In line with these findings, the drug screen results demonstrated that ID12 cells were very sensitive to afatinib, dacomitinib and neratinib, but not to the reversible ERBB2 inhibitor lapatinib. Finally, the drug screen data identified ID2_FT2 as one of the most sensitive patient spheroids to EGFR inhibitors. The genomic sequencing data of this sample did not reveal any alteration that could predict this hypersensitivity (Supplementary Data 1). Of interest, patients’ spheroid responses to EGFR/ERBB2 inhibitors followed a similar trend to their responses to the Bruton’s tyrosine kinase (BTK) inhibitor ibrutinib, (Fig. 4a, b), suggesting the inhibition by ibrutinib of the ERBB family kinases in addition to the BTK, as reported elsewhere^33,34^.

### Drug screen results of mCRC patient-derived spheroids recapitulate patients’ clinical responses to anti-EGFR therapy

**Fig. 5:**
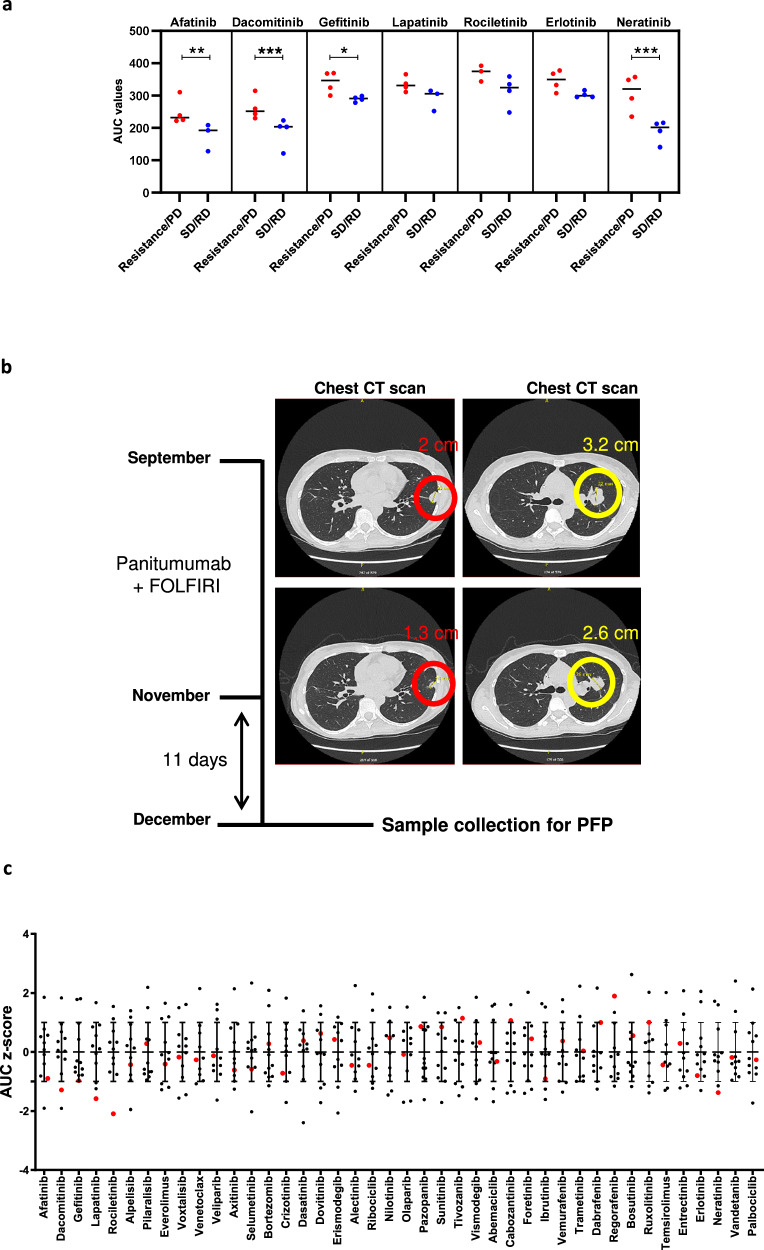
Anti-EGFR drug screen results of mCRC patient-derived spheroids are in line with patients’ clinical responses to EGFR inhibitor therapy. **a** mCRC patients who received an anti-EGFR monoclonal antibody as last treatment prior to, or first treatment after sample collection and patients bearing a genetic marker of resistance to anti-EGFR therapy were clustered into two groups: patients that exhibited a progressive disease (PD) to the anti-EGFR treatment were combined with the genetically-resistant ones to form the resistance/PD group. Patients who showed a stable (SD) or regressive disease (RD) as first clinical response after anti-EGFR therapy formed the SD/RD group. AUC values from the anti-EGFR drug screen were compared between the two clinical response groups. *P < 0.05; **P < 0.01; ***P < 0.001 using 3-way ANOVA with Tukey’s post-hoc analysis. **b** Chest computed tomography (CT) scans of patient ID2 showing regression of the disease under panitumumab+FOLFIRI. The CT scans performed in September reveal the presence of 2 metastases: a 3.2-cm tumor in the left upper lobe and a 2-cm pleural metastasis in the left lower lobe of the lung. In November of the same year, the CT scan images demonstrate the regression of the disease with the shrinkage of the metastases to 2.6 cm and 1.3 cm, respectively. Eleven days later, the sample was collected from the lung for inclusion in the study. **c** Drug sensitivity profiles of mCRC patient-derived spheroids. Patient-derived spheroids (*n* = 12 patients) underwent drug sensitivity screening to 42 compounds. The scatter plot shows the normalized AUCs (z-scores) of the patient spheroids for the indicated drugs, their mean and standard deviation. Each dot represents a patient. The red dots correspond to AUC z-scores of ID2_FT2 spheroids recapitulating the clinical sensitivity of patient ID2 tumor to anti-EGFR therapy.

To check whether anti-EGFR drug sensitivities ex vivo reflect patients’ clinical responses, we compared the AUC values of the dose-response curves obtained with the 7 anti-EGFR compounds of the drug screen with the clinical responses to anti-EGFR therapy of the patients as provided by the radiological clinical reports (Table 1). Only patient responses to the last therapy before, or the first therapy after sample collection were considered, since tumors can undergo significant changes over time and in response to anticancer therapy. Accordingly, patients ID1, ID5, ID8 and ID12 were excluded from this analysis. Patients ID3, ID4 and ID9 bearing known resistance mutations to single-agent anti-EGFR therapy were considered *de facto* resistant to this treatment. Patient ID7 experienced a progressive disease under FOLFIRI combined to panitumumab just before sample collection. Hence, patients ID3, ID4, ID9 and ID7 were clustered into the genomic-based resistance/progressive disease group. Patients ID2, ID6, ID10 and ID11 showed signs of stable or regressive disease when exposed to FOLFIRI/FOLFOX and panitumumab. Therefore, these 4 patients formed the stable/regressive disease group. We compared the AUC values obtained with the anti-EGFR compounds in both tumor response groups. As shown in Fig. 5a, the AUC-based anti-EGFR drug responses clearly distinguished between the two clinical response groups (*p* = 1.5 × 10^−11^, 3-way ANOVA). In particular, statistically significant differences were observed with afatinib (*p* = 0.006), dacomitinib (*p* = 0.0005), gefitinib (*p* = 0.03) and neratinib (*p* = 9.0 × 10^−8^). These data suggest that personalized functional profiling can predict clinical tumor response to anti-EGFR therapy in mCRC.

**Table. 1:** Clinical information of mCRC patients

Patient identification number	Gender	Age at collection time (y)	Anatomic site of collected tissue	History of sequential anticancer drug treatments prior to sample collection	Last anticancer drug therapy prior to sample collection	Clinical response to the last treatment prior to sample collection	First anticancer drug therapy after sample collection	Clinical response to the first treatment after sample collection
ID1	M	55	Liver	FOLFOX+Panitumumab; Folinic acid+5-FU; FOLFIRI	FOLFIRI	Progressive disease	FOLFIRI+Bevacizumab	Stable/regressive disease
ID2	F	41	Lung	FOLFOX; Capecitabine+Bevacizumab; Capecitabine+Aflibercept; FOLFIRI+Panitumumab	FOLFIRI+Panitumumab	Regressive disease	FOLFIRI+Neratinib	Progressive disease
ID3	F	59	Mesenteric lymph node	FOLFIRINOX; FOLFOX; Encorafenib+Cetuximab	Encorafenib+Cetuximab	Progressive disease	FOLFIRI	Progressive disease
ID4	M	60	Lung	FOLFOX; FOLFIRI+Aflibercept; TAS-102+Ramucirumab; FOLFOX	FOLFOX	N.A.^a^	FOLFOX	Progressive disease
ID5	M	57	Abdominal muscular layer	FOLFOX; Mitomycin C; FOLFIRI+Panitumumab; FOLFIRI+Bevacizumab	FOLFIRI+Bevacizumab	Stable disease	FOLFIRI+Bevacizumab	Regressive disease
ID6	F	57	Liver	None	None	N.A.	FOLFOX+Panitumumab	Stable/regressive disease
ID7	F	57	Lung	Capecitabine; FOLFOX; FOLFIRI+Panitumumab	FOLFIRI+Panitumumab	Progressive disease	FOLFIRI+Panitumumab	Progressive disease
ID8	M	48	Hypochondrium	None	None	N.A.	Mitomycin C^b^+Pembrolizumab	Stable disease
ID9	F	61	Liver	FOLFOX+Bevacizumab; FOLFIRI+Bevacizumab; FOLFIRI	FOLFIRI	Progressive disease	FOLFIRI	Progressive disease
ID10	F	50	Colon	FOLFOX	FOLFOX	N.A.^c^	FOLFOX+Panitumumab	Regressive disease
ID11	M	72	Liver	FOLFOX+Panitumumab	FOLFOX+Panitumumab	Regressive disease	Capecitabine	Progressive disease
ID12	F	32	Abdominal wall	FOLFOX+Panitumumab; Capecitabine	Capecitabine	Progressive disease	FOLFIRI+Bevacizumab	Progressive disease

*FOLFOX* folinic acid + fluorouracil + oxaliplatin, *FOLFIRI* folinic acid + fluorouracil + irinotecan, *FOLFIRINOX* folinic acid + fluorouracil + irinotecan + oxaliplatin, *N.A.* not applicable.

^a^FOLFOX treatment started one month before sample collection.

^b^Mitomycin C-based hyperthermic intraperitoneal chemotherapy.

^c^Sample collected 2 days before the start of the treatment.

In the next paragraph, we will develop the results obtained with patient ID2 and corresponding spheroids, as an interesting case report revealing sensitivity to anti-EGFR therapy at the time of sample collection followed by the development of drug resistance.

After several treatment lines, patient ID2 received panitumumab combined to FOLFIRI (Table 1). A thoraco-abdominal computed tomography (CT) scan performed 11 days before sample collection for PFP showed evidence of a regressive disease under this treatment combination, as shown by the shrinkage of the pulmonary metastases (in the left upper lobe, a metastasis shrank from 3.2 cm to 2.6 cm and a pleural metastasis in the left lower lobe of the lung shrank from 2 cm to 1.3 cm) (Fig. 5b).

The drug screen results of ID2_FT2 spheroids showing the hypersensitivity of this sample to all the EGFR/ERBB2 inhibitors tested (Fig. 4) were in accordance with the responsiveness of this patient’s tumor to anti-EGFR therapy. The AUC z-score values classified ID2_FT2 in the ~16% most sensitive patient spheroids to dacomitinib (AUC z-score = −1.2890), gefitinib (AUC z-score = −0.9724), lapatinib (AUC z-score = −1.5799), rociletinib (AUC z-score = −2.0930) and neratinib (AUC z-score = −1.3763). The hypersensitivity of this sample was specific to this drug family and did not result from a general favorable drug sensitivity profile of the sample (Fig. 5c).

Thirty-three days after the PFP results were issued, the patient started neratinib treatment in addition to FOLFIRI. Unfortunately, a CT scan performed forty-eight days later revealed a progressive pulmonary disease, suggesting the development of drug resistance. Interestingly, a low-frequency KRAS p.Q61H mutation (VAF = 2.61%) detected in the ID2 parental tumor was not identified in the corresponding spheroids, reflecting tumor heterogeneity and clonal evolution (Fig. 3 and Supplementary Data 1). The presence of this mutation has been associated with acquired resistance to anti-EGFR agents in CRC^35,36^ and may explain the progression of the disease under neratinib treatment. These findings provide evidence that genomic and functional profiling data are complementary in providing treatment guidance.

### Drug screening of mCRC patient-derived spheroids fails to mirror clinical response to anti-angiogenic therapy

**Fig. 6:**
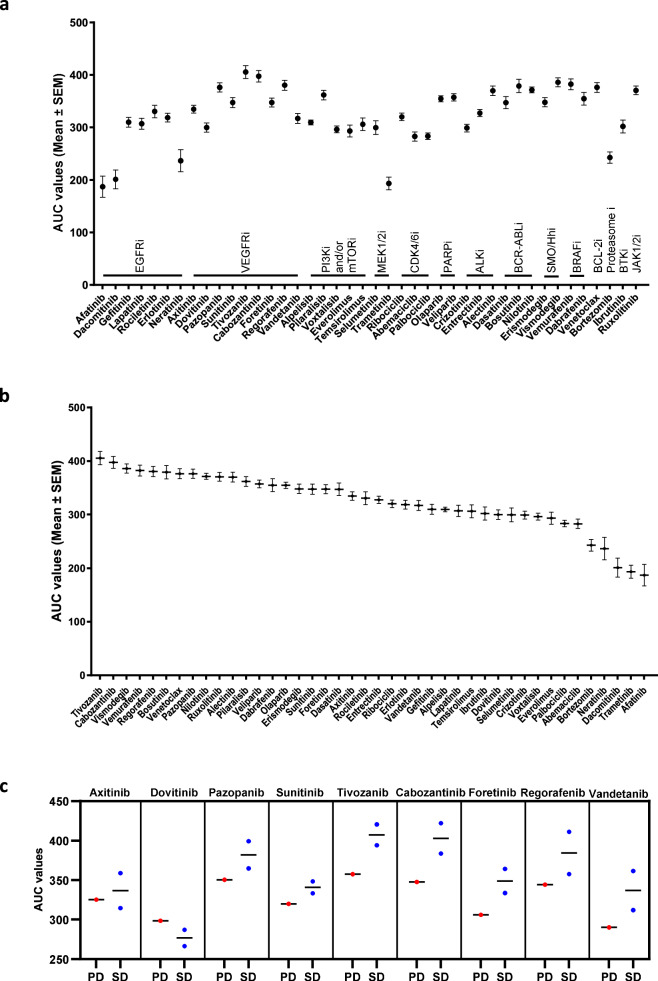
VEGFR inhibitors exhibit limited in vitro activity and show poor relation with clinical response. The AUC values of the mCRC patient-derived spheroid responses to the 42-drug library are displayed by drug family (**a**) and in descending order (**b**). In (**c**), the AUC values of VEGFR inhibitors illustrate the discordance between the drug screen results and the clinical responses. The mCRC spheroids were drug-screened as described in Methods. The AUC values were obtained from the percentages of viability using GraphPad Prism 10.3.1. The scatterplots in (**a**, **b**) show the mean AUC values ± standard error of the mean (SEM) (*n* = 10–12) obtained with each drug. The scatterplots in (**c**) show the AUC values and their median in the progressive-disease (PD) and the stable-disease (SD) clinical response groups.

Next, we aimed to know whether the drug screen results recapitulate clinical responses to anti-angiogenic therapy. The comparison of patients’ spheroid AUC values between drug families revealed a strong effect of the drug family in the obtained results (*p* < 2 × 10^−16^, 2-way ANOVA). Overall, most VEGFR inhibitors used in the drug screen exhibited no or mild cell viability inhibition in mCRC spheroids as suggested by the relatively high AUC values obtained from the dose-response curves when compared to other drug families (Fig. 6a). Significant differences were observed between the VEGFR inhibitor family and each of the following drug families: proteasome inhibitor (*p* = 1.07 × 10^−13^), MEK1/2 inhibitor (*p* = 1.44 × 10^−13^), EGFR inhibitor (*p* = 2.09 × 10^−11^), CDK4/6 inhibitor (*p* = 0.38 × 10^−5^), BTK inhibitor (*p* = 0.12 × 10^−3^) and PI3K and/or mTOR inhibitor (*p* = 0.01). Of note, the two highest mean AUC values in the drug screen corresponded to cell responses to VEGFR inhibitors (tivozanib and cabozantinib) (Fig. 6b). Based on patients’ responses to the anti-angiogenic drug received as the last treatment prior to, or as the first treatment after sample collection, the patients can be clustered into 2 groups: a progressive disease group comprising patient ID12 and a stable-disease group consisting of patients ID1 and ID5. All the other patients were excluded from this analysis either because their anti-angiogenic treatment was not immediately following or preceding sample collection or because they did not receive such therapy before their inclusion in the study. Next, we compared the AUC values obtained with the 9 anti-VEGFR compounds in both tumor response groups. Given the small sample size (*n* = 3), no reliable conclusions could be drawn. Yet, our data did not show higher AUC values, suggestive of a more resistant profile, for the progressive-disease sample ID12 (Fig. 6c). This observation implies that the absence of blood vessels in ex vivo 3D models, such as the spheroids used in this study, may impair the evaluation of anti-angiogenic drug responses in mCRC.

### Personalized drug screening confirms resistance to BRAF-targeted therapy in the BRAF p.V600E-mutated ID3_FT1 sample

Patient ID3, diagnosed with a BRAF p.V600E-mutated mCRC, was treated with FOLFIRINOX followed by FOLFOX, then received encorafenib in combination with cetuximab for ~5 months. During the course of the targeted therapy, a cerebral magnetic resonance imaging (MRI) showed a size increase of a cerebellar metastasis from 6.5 mm to 18 mm in diameter and a thoraco-abdomino-pelvic CT scan revealed new liver metastases (Supplementary Fig. 3a) and size-progressive retroperitoneal and mesenteric lymph nodes. A metastatic lymph node excision was performed 13 days later and a specimen of the tumor was collected for PFP. The drug screen performed on ID3_FT1 cells highlighted the overall drug resistance of this patient sample (Supplementary Fig. 3b). In particular, AUC z-scores >1 (Supplementary Fig. 3b) and absence of a dose-response effect (Supplementary Fig. 3c) confirmed resistance to the BRAF inhibitors, vemurafenib and dabrafenib, and to regorafenib that targets, among others, BRAF p.V600E.

Since patient ID3 received encorafenib in combination with cetuximab as the last treatment prior to sample collection, we evaluated the sensitivity of ID3_FT1 cells to this combination to determine whether the in vitro response was concordant with the clinically observed resistance. As shown in Supplementary Fig. 3d, dose-response curves (DRCs) for ID3_FT1 cells treated for 3 days with encorafenib alone or in combination with cetuximab overlapped with those of the other samples classified as clinically non-responders to this regimen (this combination is clinically indicated only for BRAF V600E-mutant mCRC and not for BRAF wild-type disease).

Because cetuximab is a monoclonal antibody whose activity in patients partly depends on immune-related mechanisms^37^, we next sought to exclude the possibility that the absence of immune cells in our spheroid model compromised its activity. To this end, ID3_FT1 cells were treated for 3 days with encorafenib in combination with the small-molecule EGFR inhibitors gefitinib or erlotinib. The resulting DRCs showed no evidence of enhanced sensitivity, as they overlapped with those of other samples and were associated with relatively high IC_50_ values compared with other spheroid samples (Supplementary Fig. 3d). Collectively, these results confirm the lack of sensitivity of ID3_FT1 cells to the combined BRAF p.V600E and EGFR inhibition, consistent with the clinically documented disease progression of patient ID3 during treatment with encorafenib in combination with cetuximab (Table 1).

### Personalized drug screening of mCRC patient-derived spheroids reveals variable responses to standard-of-care (SOC) treatments

To study whether our PFP strategy can identify sensitivity profiles to standard-of-care (SOC) regimens, eight spheroid samples were exposed, for 3 days, to increasing concentrations of 5-fluorouracil (5-FU), SN-38 (the active metabolite of irinotecan), oxaliplatin, 5-FU+oxaliplatin, 5-FU+SN-38, 5-FU+SN-38+oxaliplatin, 5-FU+SN-38+cetuximab and 5-FU+oxaliplatin+cetuximab, in an attempt to mimic the clinical regimens received by the patients.

**Fig. 7:**
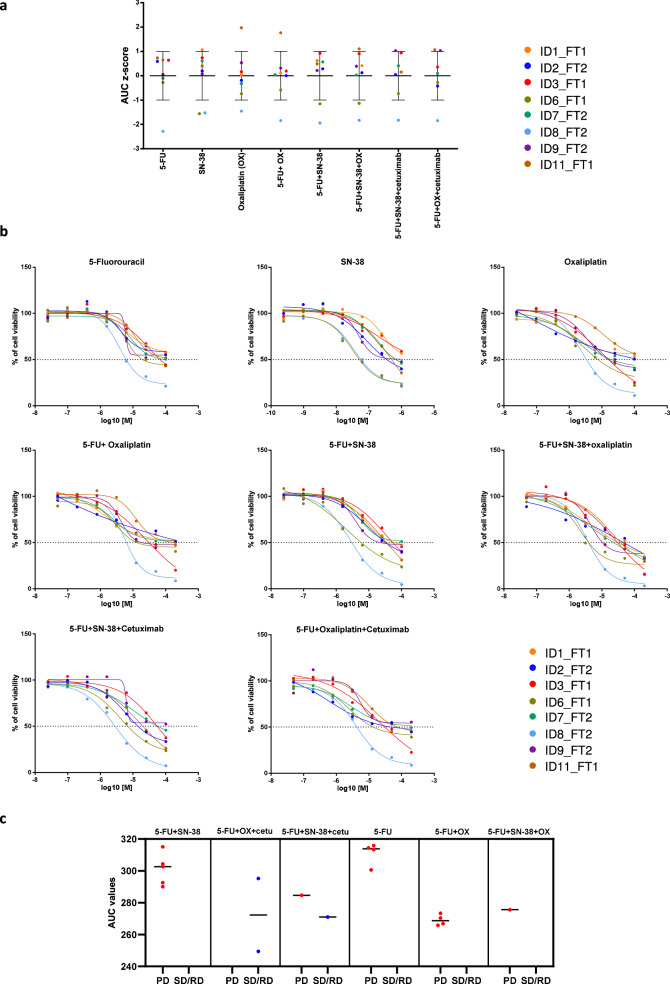
Personalized drug screening of mCRC patient-derived spheroids reveals variable responses to standard-of-care (SOC) treatments. mCRC spheroids (*n* = 7–8) were subjected to drug screening with the SOC agents during 3 days, as described in Methods. The data from the dose-response curves generated with the indicated drugs and drug combinations are presented. The scatterplot in (**a**) shows the individual AUC z-scores with their mean and standard deviation per treatment. Each patient-derived spheroid sample is represented by a dot. In (**b**), the corresponding dose-response curves are displayed. The graphs show the means (*n* = 2–4) and the fitted sigmoidal curve of each sample incubated with increasing concentrations of the indicated drugs or drug combinations (fixed ratio). In (**c**), The scatterplots show the AUC values of the DRCs and their median, in the progressive-disease (PD) and the stable-disease/regressive disease (SD/RD) clinical response groups. mCRC patients were clustered into these two groups based on their clinical response to any treatment line prior to sample collection and to the first treatment line after sample collection. When patients showed a progressive disease under chemotherapy+targeted therapy, they were classified as resistant to the chemotherapy as well as to the combination regimen, and therefore included in the corresponding PD groups. However, when patients showed a SD/RD under chemotherapy+targeted therapy, they were classified in the SD/RD group for the combination regimen only. Any clinical treatment with panitumumab was treated as cetuximab for analysis. Responses to capecitabine were considered as responses to 5-FU. Clinical treatments with FOLFIRI, FOLFOX and FOLFIRINOX were compared to drug screen results with 5-FU+SN-38, 5-FU+oxaliplatin and 5-FU+SN-38+oxaliplatin, respectively.

Among all the spheroids tested, ID8_FT2 and ID6_FT1 displayed overall the lowest AUC z-scores and the smallest IC_50_ towards SOC treatments (Fig. 7a, b). Interestingly, ID8_FT2 and ID6_FT1 were the only spheroids generated from chemotherapy-naïve tumors. Since the corresponding tumors haven’t been exposed to a previous selective drug pressure in the clinic, the absence of a secondary drug resistance may explain, at least partly, the higher sensitivity of their corresponding spheroids to SOC drugs.

In the present study, ID8 was the only MSI-high, MMR-deficient tumor, exhibiting a pathogenic truncating mutation in MLH1. MSI-high status in CRC has been associated with resistance to 5-FU-based chemotherapy^38,39^. Nonetheless, ID8_FT2 spheroids displayed the highest sensitivity to 5-FU (Fig. 7a, b).

ID8 spheroids were also the most sensitive spheroids to SN-38. This result is concordant with the demonstrated relationship between CRC responsiveness to irinotecan and high MSI^40^.

The majority of the patients included in the present study had already received several treatment lines and subsequently developed resistance to these treatments prior to sample collection. Consequently, the identification of responders and non-responders to SOC drugs is very challenging as the cohort largely consists of non-responders with progressive disease. Because our profiling strategy relies on comparing patient responses per drug, a balanced distribution of clinically responsive and non-responsive cases is essential. However, this condition is not met in the present cohort (Fig. 7c). Therefore, any attempt to predict or identify clinical responses based on SOC drug screening data would be misleading. Nevertheless, several spheroid drug response results appear to be consistent with the corresponding patients’ clinical outcomes (in this analysis, and in response to a reviewer’s request, we considered clinical responses to any treatment line (and not only to the last treatment line) prior to sample collection and to the first treatment line after sample collection). In particular, ID3_FT1 tended to display low sensitivity to 5-FU+SN-38+oxaliplatin (the second highest AUC value) and to 5-FU+SN-38 (the highest AUC value), in line with the clinical progression of the disease under FOLFIRINOX and FOLFIRI (Table 1). In addition, ID9_FT2 appeared as one of the most resistant spheroids to 5-FU+SN-38+cetuximab and to 5-FU+oxaliplatin+cetuximab, as indicated by high AUC values and elevated IC_50_ values. Given that the ID9 tumor harbors KRAS p.G12V mutation, these findings are consistent with the intrinsic resistance of this sample to anti-EGFR therapies and with the patient’s clinical disease progression under FOLFOX and FOLFIRI. (Table 1). In contrast, ID11_FT1 spheroids exhibited low sensitivity to oxaliplatin-based combinations, as indicated by the highest AUC values in the corresponding drug screen data, which contrasted with the clinical disease regression observed after six cycles of FOLFOX plus panitumumab (Fig. 7a, b and Table 1). Of note, following this treatment, patient ID11 underwent approximately one month of preoperative chemoradiation with capecitabine, a 5-FU prodrug, during which disease progression was observed. The patient was subsequently treated with FOLFOX for two months, until further evidence of disease progression emerged. Interestingly, the drug screening data indicated low sensitivity to oxaliplatin-based regimens, potentially reflecting imminent clinical resistance, though it is not yet clear whether this is truly predictive or merely coincidental.

In conclusion, personalized drug screening revealed substantial variability in mCRC responses to SOC treatments; however, its predictive value for these regimens remains to be established.

### Patient-derived spheroids display cellular heterogeneity in stemness and differentiation markers, without a clear association between drug resistance and the proportion of cancer stem cells

Evidence suggests that cancer stem cells (CSCs) are involved not only in cancer development but also in metastasis and drug resistance^41^. To assess the presence of a population with CSC traits in patient-derived spheroids and to evaluate its potential contribution to drug resistance, we analyzed the expression of CD44 and CD133 markers commonly expressed in CSCs, by flow cytometry in nine patient-derived spheroid samples. Cells expressing CD44 and/or CD133 were classified as CSCs/CSC-like cells. As shown in Supplementary Table 4, the proportion of CSCs/CSC-like cells varied markedly among spheroids, ranging from an almost complete absence in ID8_FT2 (0.07%) to 61.9% in ID7_FT2. With the exception of ID7_FT2 in which the CD44+/CD133+ double-positive population accounted for 16.7% of the cells, this population represented less than 5% of cells in all other samples, consistent with the reported rarity of CD44+/CD133+ cells in colorectal cancer^42^. Flow cytometry plots of CD44+/CD133−, CD44−/CD133+, CD44+/CD133+ and CD44−/CD133− cell populations are shown in Supplementary Fig. 4a.

To investigate the contribution of the CSC population to drug resistance of patient-derived spheroids, we analyzed correlations between AUC values obtained from the 42-drug library and the SOC drugs, and the percentages of CD44+, CD133+ and CD44+/CD133+ cells. No correlations were observed between AUC values from the 42-drug library and the percentages of CD44+, CD133+ or CD44+/CD133+ cells (Supplementary Fig. 5). Similarly, AUC values from the SOC drugs showed no correlation with the percentages of CD44+ or CD44+/CD133+ cells. Significant correlations were detected only between some SOC drug AUC values and the percentage of CD133+ cells, consistent with previous reports^43^. Results of the correlation analysis were confirmed by using linear mixed-effects models (ANCOVA) (after Benjamini–Hochberg’s corrections).

CDX2 is an intestinal differentiation marker. Loss of CDX2 expression reflects poor differentiation and an aggressive tumor phenotype^44^. To examine whether patient spheroids support the presence of cells at multiple differentiation stages, we have also analyzed the expression of CDX2 in the nine patient-derived spheroids. As shown in Supplementary Table 4 and in Supplementary Fig. 4b, CDX2-positive cells were present at variable proportions in patient spheroids. ID8_FT2 contained the smallest percentage of CDX2-positive cells, in line with the reported loss of CDX2 expression in microsatellite-unstable tumors^44^, whereas CDX2-positive cells represented 93.2% of ID1_FT1 cells. Positivity for both CDX2 and either CD44 or CD133 was also observed, indicating the presence of a broad spectrum of cell populations within patient-derived spheroids.

## Discussion

In this manuscript, we describe our personalized functional profiling approach in mCRC using patient-derived spheroids, provide a technical validation of the method and discuss its utility in the identification of personalized treatment options as well as in the prediction of drug resistance. In particular, the personalized drug screening identified mCRC tumors exhibiting a genetic marker of resistance to anti-EGFR therapy, highlighted potential candidates for anti-ERBB2 treatment and demonstrated its value in predicting clinical responses to anti-EGFR therapy.

Turnaround time is a critical determinant for the clinical implementation of 3D model-based functional precision oncology. In the present study, the spheroid generation success rate was 63%, which is comparable to rates reported in previous studies^45,46^. When fresh spheroids were used, the median time from sample collection to cell printing was 34 days, and the median overall turnaround time to release of drug screening results was 55.5 days. Excluding two cases that required screening repetition due to technical issues, the median turnaround time was reduced to 39 days, highlighting the importance of technical robustness for timely result delivery. These timelines are comparable to those reported in other 3D-model guided precision oncology studies. In the SENSOR trial, drug sensitivity data obtained from patient-derived tumor models were generated within 10 weeks of biopsy^45^. Similarly, Cartry et al. demonstrated the feasibility of patient-derived organoid-based drug screening in advanced colorectal cancer with a median turnaround time of 6 weeks while testing a panel of 25 FDA-approved drugs and achieving clinical concordance in retrospective analyses^46^.

Our findings are in line with previous reports demonstrating that patient-derived tumor models recapitulate morphological and genetic features of the parental tissue and describing their successful use in proof-of-concept drug screens^6,19,47^. In this regard, Van de Wetering et al. carried out a high-throughput (HTP) drug screen analysis using tumor organoids from 19 previously untreated CRC patients and detected gene-drug associations, as shown by the sensitivity to the inhibition of Wnt secretion of an organoid culture bearing a mutation in the Wnt feedback regulator RNF43^19^. Also, Pauli et al. subjected 4 tumor organoid samples from late-stage cancers to HTP drug screens and identified effective targeted agents and optimal combination therapies^6^. Vlachogiannis et al. compared clinical responses in gastrointestinal cancer patients with drug response data gathered in corresponding patient-derived organoids (PDO) and showed that PDO recapitulate patient responses to paclitaxel, to 5-FU+ cisplatin, to cetuximab and to TAS-102^20^. We and collaborators have reported the use of patient-derived spheroids in drug screens that permitted the identification of hit drugs in glioblastoma and metastatic lung atypical carcinoid^48,49^.

Mao et al. performed a colorectal cancer organoid-based drug screen using 34 preselected drugs in addition to regorafenib and 5-FU (considered as positive control drugs) at different concentration ranges over a 5-day incubation time^16^. By assessing the growth rate inhibition by 50% (GR_50_), the authors demonstrated that romidepsin, trametinib and bortezomib displayed the highest growth inhibitory effects. In the present study, we have tested the cell viability inhibition of 42 drugs (6 drugs overlapped with those reported in the study of Mao et al.) on mCRC spheroids maintained with the drugs for 5 days. We have observed, similarly to Mao et al., that trametinib and bortezomib were among the most powerful drugs in inhibiting cell viability (Fig. 6b). Moreover, in both studies, regorafenib and bosutinib were classified among the drugs that exhibited the lowest inhibitory effects. Despite the different 3D cell models (organoids versus spheroids), readouts (ATP measurement versus calcein-stained cell area) and metrics chosen to evaluate drug responses (GR_50_ versus AUC), the concordance of the results in Mao et al. study^16^ and in our work suggests the reliability of our drug screen approach.

Here, we show that mCRC patient-derived spheroids are robust models for recapitulating key histopathological features and genetic landscape of matched patient’s tumor. In addition, we demonstrate the utility of biobanked spheroids as a platform for personalized medicine research, since cryopreservation did not significantly alter these characteristics nor the drug profiling results. Moreover, we identify several genotype-drug response matches that validate the robustness of our PFP approach. In particular, the drug screen results confirm the resistance to EGFR inhibition of KRAS- and BRAF-mutated tumors and highlight the sensitivity of ERBB3 p.V104M- and ERBB2 p.G776delinsVC-mutated tumors to dual EGFR/ERBB2 inhibitors and irreversible ERBB2 inhibitors, respectively. Whereas RAS pathway mutations are strong predictive biomarkers for resistance to anti-EGFR therapy in mCRC, there is no validated biomarker to select CRC patients who would most likely respond to this therapy^50^. In this context, our PFP approach emerges as a promising alternative to address this therapeutic gap. Notably, our drug screen results matched patients’ clinical responses to anti-EGFR therapy, distinguishing resistant patients from those experiencing a stable or regressive disease. In particular, ID2 tumor cells, collected from a panitumumab-sensitive patient, were highly sensitive to EGFR inhibition as shown by the drug screen results. The secondary clinical resistance to neratinib developed by patient ID2 several weeks after the completion of the drug screen is in line with the documented time-limited clinical benefit of anti-EGFR drugs in responder patients^51^. The identification, in the patient sample but not in the spheroids, of KRAS p.Q61H mutation at a low VAF (2.61%) may suggest that, under neratinib treatment, the prevalence of KRAS mutant clones increased, resulting in disease progression. The purpose of PFP is to release drug screen results in time frames compatible with cancer patient management; in this race against time, genomic data are complementary to PFP results in predicting drug responsiveness.

An interesting finding from our drug screen is that the response to ibrutinib matched patient’s response to EGFR/ERBB2 inhibitors. This observation, likely due to ibrutinib targeting EGFR and ERBB2^34^, further supports the validity of our drug screen data.

Of note, the drug screen results did not correlate with the patients’ clinical responses to antiangiogenic agents. Overall, anti-VEGFR compounds did not exhibit a clear viability inhibition activity in mCRC spheroids. This observation cannot be solely explained by the different relevant concentrations of bioactivity of anti-VEGFR molecules that would necessitate adjusting the concentration ranges of the drugs. For example, plasma AUC, namely the integration of plasma drug exposure over time, of cabozantinib and lapatinib are comparable (41.6 µg.hr/mL and 36.2 µg.hr/mL, respectively) and their previously suggested highest concentrations in drug screens are similar^52,53^. Nevertheless, their activities in our drug screen were significantly different. Likewise, we did not detect any effect of regorafenib, an antiangiogenic agent used in the treatment of mCRC patients, on the viability of patient-derived spheroids ex vivo, in line with previous reports suggesting that response to regorafenib is dependent on the presence of a vascular system^16,20^.

In this study, the 42-drug library consisted mostly of Food and Drug Administration (FDA)/European Medicines Agency (EMA)-approved drugs that were tested only as monotherapy in the screen assay. Such design can indeed lead to the identification of potential hit drugs with promising antitumour activities in vitro, but with the risk of not demonstrating a single-agent efficacy in the clinic. In particular, molecules with EGFR inhibition activity, namely gefitinib, erlotinib and vandetanib, elicited anti-tumor response in colorectal cancer models in vitro or in patient-derived xenografts but in some instances failed to show a beneficial effect when administered as single-agents to the patients^54–59^. In addition, BRAF inhibitors administered as monotherapy are not effective in achieving clinical benefit in BRAF-mutated mCRC patients (response rates of ~5%) and the addition of an anti-EGFR agent (such as cetuximab) may synergize with the BRAF inhibitor to alleviate tumor growth^60^. Our drug screen data obtained with the combined inhibition of BRAF p.V600E and EGFR confirm the results obtained with BRAF inhibitors alone and correlate with the clinically documented ID3 patient resistance to encorafenib in combination with cetuximab.

We also evaluated whether our PFP approach could predict tumor responses to standard-of-care (SOC) therapies. Patient-derived spheroids exhibited heterogeneous responses to chemotherapy regimens, and only a subset of drug screening results correlated with clinical outcomes or tumor genetic features. Notably, spheroids derived from chemotherapy-naïve tumors showed the highest sensitivity to SOC drugs, consistent with the absence of acquired resistance mechanisms and in agreement with previous observations by Papaccio et al.^61^. As expected, MSI-high patient-derived spheroids (ID8_FT2) displayed increased sensitivity to irinotecan. This finding is consistent with prior reports demonstrating enhanced irinotecan activity in MSI-high compared with MSS colorectal cancer, attributable to deficient mismatch repair (MMR) and the consequent accumulation of irinotecan-induced DNA double-strand breaks^40,62^. Unexpectedly, ID8_FT2 spheroids were also the most sensitive to 5-fluorouracil (5-FU), despite the well-documented resistance of MSI-high CRC to 5-FU-based chemotherapy^63^. However, selected studies have reported improved survival in subsets of MSI-high patients treated with adjuvant 5-FU-based regimens^64^. This paradoxical sensitivity has been attributed to MMR-independent mechanisms, potentially involving intact p53 signaling, as most MSI-high tumors retain wild-type TP53, whereas MSI-low/MSS tumors frequently harbor TP53 mutations. Consequently, MSI-high, TP53 wild-type tumors may exhibit increased responsiveness to 5-FU relative to MSI-low/MSS, TP53-mutant tumors^65,66^. In our cohort, ID8 was the only tumor with wild-type TP53, which may contribute to its enhanced sensitivity to 5-FU.

In contrast, the resistance of ID11_FT1 spheroids to oxaliplatin-based treatments did not align with the clinically observed regressive disease. Ooft et al. have already reported the limited ability of patient-derived organoid/screening system to accurately recapitulate clinical responses to fluorouracil and oxaliplatin combinations, compared with irinotecan-based therapies^67^.

Nevertheless, our drug screening data revealed that KRAS- and BRAF-mutant patient-derived spheroids were among the most resistant to chemotherapy combined with cetuximab. This finding aligns with the clinical progression of these patients under 5-FU-based regimens and with the known intrinsic resistance of KRAS- and BRAF- mutant tumors to anti-EGFR therapies. Importantly, our patient cohort predominantly consisted of non-responders with progressive disease, which renders any prediction of clinical response very challenging.

The drug screening results with SOC treatments are highly encouraging; however, a more balanced cohort encompassing a broader range of clinical responses will be needed to establish clearer conclusions.

Patient-derived spheroids have been widely used as in vitro models to enrich cancer stem cells (CSCs) or cells with stem cell-related features, thereby enabling the assessment of CSC-associated characteristics in solid tumors^68^. Cancer cell stemness has been implicated in chemoresistance and metastasis, and multiple studies report that CSCs display greater resistance to chemotherapeutic agents than the bulk tumor cell population^69,70^. These observations raise the question of whether drug screening outcomes are primarily influenced by the proportion of CSCs within patient-derived spheroids. Notably, our patient-derived spheroids comprised a heterogeneous population of CSCs and more differentiated tumor cells. Our correlation analysis did not support a direct association between drug resistance profiles and CSC enrichment in patient-derived spheroids, suggesting that drug responses were predominantly driven by the intrinsic characteristics of the original tumor cells rather than by technical bias introduced during spheroid culture.

While further validation in larger cohorts remains necessary to fully validate the translational potential of our personalized functional profiling approach, the data presented here indicate that ex vivo assessment of chemosensitivity and chemoresistance via PFP can serve as a predictive tool for clinical drug response in mCRC. This enables the identification of patients most likely to benefit from specific therapies as well as those at high risk of disease progression.

Our PFP platform supports high-throughput drug screening using patient-derived 3D models and delivers results within clinically compatible time frames. The automated printing in pillar plates, adaptable to different matrices, minimizes cell loss and preserves the 3D structures throughout experimental steps. In addition, the platform requires only small amounts of starting material, is amenable to quantitative analysis and remains user-friendly. Ultimately, the integration of PFP into clinical workflows has the potential to significantly advance precision cancer medicine.

This study has several limitations that should be acknowledged. First, while the observed turnaround time is comparable to that reported in other organoid-based clinical trials, it may still be too long to meaningfully inform treatment decisions in patients with rapidly progressing disease. In this pilot study, the use of a relatively broad drug library comprising 42 compounds increased cell requirements and consequently prolonged screening timelines. Based on the experience gained, we are now refining our approach by defining more focused drug panels that prioritize agents according to tumor type and clinical relevance, while excluding compounds with a low likelihood of activity (such as VEGFR inhibitors in non-vascularized models). Implementation of such a focused panel is expected to reduce turnaround time and improve clinical feasibility in subsequent studies.

Second, the cohort was small and largely enriched for patients who had already progressed on chemotherapy. As a result, larger and more representative cohorts spanning a broader range of clinical responses will be needed to more rigorously assess the predictive performance of the approach.

Third, the patient-derived spheroid model used in this study does not recapitulate the tumor microenvironment (TME). To address this limitation, we are currently developing mCRC organoid models that incorporate stromal cells, with the aim of capturing TME-mediated effects on drug response and enabling the evaluation of immune checkpoint inhibitors and TME-targeting molecules^17,71^.

Finally, although AUC-based z-score normalization enables a robust ranking of drug sensitivity within a cohort, validation in larger, independent cohorts will be required before this approach can be used as a clinical decision tool. Importantly, the present study was designed as a pilot investigation, and the lessons learned regarding drug panel optimization and workflow efficiency will be directly implemented in the design of subsequent studies currently in preparation.

## Methods

### Study cohort

The subject cohort consisted of twelve metastatic colorectal cancer patients followed at Zithaklinik-Hôpitaux Robert Schuman in Luxembourg. The participants provided either tumor biopsies or resected tumor specimens, following informed consent according to the Helsinki Declaration. Table 1 summarizes patient clinical information. The study (NCT03997617) was approved by the national research ethics committee “Comité National d’Ethique de Recherche” (CNER n° 201812/04).

### Tissue processing and generation of mCRC spheroids

The tumor tissues were shipped in 5 mL of phosphate-buffered saline (PBS) on ice to the PFP laboratory and processed immediately. Briefly, each sample was transferred to a culture dish, rinsed with cold washing medium (DMEM/F12 + GlutaMAX™ (Gibco #10565018) + 1% v/v penicillin-streptomycin (Gibco #15140122) + 1% v/v amphotericin B (Gibco #15290026)) and mechanically dissociated into 0.5- to 1-mm-diameter pieces using fine dissection scissors, on ice. The minced tissue was collected in a Falcon tube, pelleted (200 g, 5 min, 4°), washed again 3 times, resuspended in the culture medium in an ultra low-attachment culture dish and placed in a 37 °C, 5% CO_2_ and 90% humidity incubator. The culture medium consisted of the washing medium supplemented with 1× StemPro™ hESC Supplement (Gibco #A10006-01), 1.8% Bovine Serum Albumin (BSA) (Gibco #A10008-01), 0.1 mM 2-Mercaptoethanol (Gibco #21985-023), 50 ng/mL EGF (R&D Systems #236-EG-01M), 50 ng/mL Noggin (PeproTech #250-38), 250 ng/mL R-Spondin-1 (PeproTech #120-38), 10 nM gastrin I (Sigma-Aldrich #G9145), 10 ng/mL FGF-10 (PeproTech #100-26), 10 ng/mL FGF-basic (Gibco #PHG0023), 50 ng/mL Wnt-3A (R&D Systems #5036-WN), 1 µM Prostaglandin E_2_ (Tocris #2296), 10 µM Y-27632 (Tocris #1254), 4 mM Nicotinamide (Sigma-Aldrich #N0636), 0.5 µM A83-01 (Tocris #2939) and 5 µM SB 202190 (Sigma-Aldrich #S7067).

The medium was changed every 2–3 days and, if needed, the spheroids were passaged using TrypLE™ Express (Gibco #12605010). When enough spheroids were generated, some of them were cryopreserved for subsequent use. The experiments were performed with either fresh or cryopreserved spheroids. In the latter case, the spheroids were labeled FT in the manuscript, followed by the number of freeze-thaw cycles. Cryopreserved spheroids were propagated in culture prior to any analysis. To minimize the risk of losing specific cell subsets or clonal populations due to increased susceptibility to freeze-thaw induced stress, spheroids subjected to a maximum of two freeze-thaw cycles were used in this study. Prolonged ex vivo expansion can predispose cells to genotypic drift and chromosomal instability, and extensive passaging of patient-derived spheroids may substantially alter the composition of the original cell population^68^. Because patient-derived spheroids are intended to model the tissue of origin, they were therefore used at low passage numbers (passage number <13, with the exception of ID11_FT1 spheroids when used in flow cytometry experiments and drug screening with SOC drugs and encorafenib (passages 19 and 20)). For patients who consented to secondary use of their samples, the derived spheroids may be shared through collaborative oncology research projects with the provider team.

### Immunohistochemistry and immunocytochemistry

Formalin-fixed paraffin-embedded (FFPE) tissue samples were prepared following the standard operating procedures of the LNS pathology laboratory. For pathological assessment of spheroids, they were first harvested, washed with PBS, pelleted (200 g, 5 min), fixed with 4% formaldehyde solution, then embedded in paraffin. Immunostaining was performed on 3-µm FFPE sections using an automated IHC stainer (Dako Omnis) and 3,3’-diaminobenzidine (DAB)-based visualisation. The sections were counterstained with haematoxylin. The antibodies against CDX2 (#IR080), cytokeratin 7 (CK7) (#IR619), mucin 2 glycoprotein (MUC2) (#IR658), cytokeratin 20 (CK20) (#IR777), Ki-67 (#GA626), MSH2 (#IR085), MSH6 (#IR086), MLH1 (#IR079) and PMS2 (#IR087) were obtained from Dako (Glostrup, Denmark).

### DNA/RNA extraction, library preparation and sequencing

DNA and RNA were extracted from fresh-frozen tumor tissues and from derived spheroids using AllPrep DNA/RNA kit (Qiagen # 80204) as recommended by the manufacturer. DNA and RNA were quantified by spectrophotometry and their purity was assessed by the A260:A280 ratio. RNA integrity was evaluated by the RNA integrity number (RIN) generated using RNA 6000 Nanochips on a 2100 Bioanalyzer (Agilent Technologies, Diegem, Belgium).

Genomic libraries were prepared from extracted DNA and RNA using the TruSight Oncology 500 High-Throughput (TSO500 HT) kit (Illumina, San Diego, CA), according to the manufacturer’s protocol. Sequencing was performed in paired-end mode (2 × 100) on an SP flow cell using the NovaSeq 6000 System. Data were processed using the TruSight Oncology 500 Combined Variant Output pipeline (version ruo-2.2.0.12) (Illumina, San Diego, CA) using manufacturer’s recommendations. Tumor Mutational Burden (TMB) and Microsatellite Instability (MSI) status were determined using pipeline’s integrated informatics. TMB was calculated as the total number of somatic mutations per megabase (mut/Mb) in the coding regions covered by the panel, including both synonymous and non-synonymous mutations, while excluding germline variants, low-confidence regions, and FFPE-induced artifacts. TMB is defined as low if TMB ≤ 5, intermediate if TMB > 5 and <10 and high if TMB ≥ 10. MSI status was assessed by analyzing 130 microsatellite loci for repeat length patterns, classifying samples as MSI-High (MSI-H), i.e. MSI > 25%, or MSI-Stable (MSS).

Subsequent interpretation of the sequenced data to identify clinically relevant variants was performed using QIAGEN Clinical Insight (QCI) Interpret software, build 9.3.1 (QIAGEN, Hilden, Germany). Common variants with an allele frequency ≥1% in gnomAD, ExAC, NHLBI ESP exomes, and the 1000 Genomes Project were excluded unless they were established pathogenic common variants. Only variants with potential effects on protein level, including structural variants associated with gene alterations, gain of function from gene fusions, copy number gains, and loss of function mutations (such as frameshift, in-frame indels, start/stop codon changes, and splice site losses) were retained. All variants of interest were further manually reviewed and classified based on ACMG (American College of Medical Genetics and Genomics) criteria to assess pathogenicity. Additionally, some variants were visually inspected using the Integrative Genomics Viewer (IGV) (Broad Institute, Cambridge, MA, USA) for further confirmation. Variant classifications shown in the oncoprint (Fig. 2) and the variant list (Supplementary Data 1) were derived from QCI at different export dates. Any discrepancies in variant classification may reflect database updates rather than true differences. The oncoprint was generated using the Oncoprinter tool in cBioPortal for Cancer Genomics. Arrows indicating TMB-High and MSI-high were added manually.

Copy number variations were called using CNVkit (version 0.9.10). Sample BAM files were processed using the batch function, with the -n flag specifying normalization to matched normal samples for accurate background correction. Targeted CNVs were identified within regions specified by the TST500C panel (-t TST500C_manifest.bed), using the human genome reference file (-f genome.fa) for alignment consistency. Post-processing, scatter plots of the copy number profiles were generated for visual inspection of CNVs. The scatter function was used with .cnr and .cns files to plot segment and region-specific CNVs across chromosomes.

### Cell printing

The spheroids were dissociated into single cells and small cell clusters using trypsin digestion. For the screening with the 42-drug library, the cells were resuspended in 0.5% alginate solution and printed as 500-nL suspension containing on average 1000 cells per condition, onto 2-mm-diameter pillars in 384-pillar plates, using the ASFA Spotter ST V5 or V6.5 (Medical & Bio Decision, Suwon-si, South Korea). After the printing, each pillar plate was left 3 min undisturbed to allow for alginate gelation, then combined with the 384-well plate containing cell culture medium for 30–45 min to remove excess BaCl_2_ coating. The printed cells were then transferred to a new 384-well plate containing 40 µL of fresh culture medium for a 24-h incubation before drug screening.

For the screening with 5-fluorouracil, SN-38, oxaliplatin, encorafenib and cetuximab, the following modifications were done: cells were resuspended in 90% ice-cold Matrigel® Growth Factor Reduced (GFR) (Corning #356231) and printed as 1.5-µL suspension. After the printing, each pillar plate was incubated (pillars up) for 5 min at 37 °C to allow the Matrigel to solidify.

### Preparation of 384-well drug assay plates and drug screening

The 42-drug library was purchased from Selleckchem and contained Food and Drug Administration (FDA)/European Medicines Agency (EMA)-approved and investigational drugs (Supplementary Table 2). Batches of 384-well assay plates containing nanoliters of concentrated drug solutions were prepared from the compound library using the Echo 550 liquid handler (Labcyte), and designed to test the drugs in four-fold and 7-point concentrations (maximal concentration = 30 µM, except for bortezomib (0.15 µM)). Foretinib (30 µM) and bortezomib (10 µM) were included as positive controls of cell death and 0.1% DMSO as a negative control to verify library drugs’ validity. Drug assay plates were sealed and stored at −80 °C until use.

The standard-of-care (SOC) drug screens were performed with 5-fluorouracil (5-FU) (Selleckchem #S1209), SN-38 (Selleckchem #S4908), oxaliplatin (MedChemExpress #HY-17371) and cetuximab (Selleckchem #A2000). The drugs were tested in four-fold and 7-point concentrations. The maximal concentrations used were 100 µM for 5-FU and oxaliplatin, 1 µM for SN-38 and 50 µg/mL for cetuximab. Drug combinations were performed at fixed ratios starting with the highest concentration of each single-agent drug: 5-FU+oxaliplatin (1:1), 5-FU+SN-38 (100:1), 5-FU+SN-38+oxaliplatin (100:1:100), 5-FU+SN-38+cetuximab (100 µM:1 µM:50 µg/mL) and 5-FU+oxaliplatin+cetuximab (100 µM:100 µM:50 µg/mL).

In drug screens where encorafenib was used, alone or in combination, it was tested at a maximal concentration of 30 µM, in four-fold and 7-point concentrations. The combinations of encorafenib+gefitinib and encorafenib+erlotinib were tested at a fixed ratio of 1:1. The combination of encorafenib+cetuximab was used at a ratio of 30 µM:50 µg/mL. Corresponding vehicle controls were included in the drug plates.

Similarly to the preparation of the 42-drug assay plates, the Echo 550 liquid handler (Labcyte) was used to dispense concentrated drug solutions of 5-FU, SN-38, encorafenib, gefitinib and erlotinib in 384-well assay plates. Oxaliplatin and cetuximab were added manually just before drug screening.

On the day of drug screening, 40 µL of culture medium were dispensed per well in the drug assay plates to reach the desired concentrations. For cetuximab treatments, EGF was removed from the culture medium. The pillar plate containing the printed cells was then “stamped” with a 384-well drug assay plate and incubated at 37 °C in a 5% CO_2_ and 90% humidity incubator for 5 days (drug screen with the 42-drug library in alginate) or for 3 days (drug screen with the SOC drugs and encorafenib in Matrigel). Each treatment condition was prepared in 2–4 replicates.

### Cell staining, image acquisition, processing and data analysis

Following drug incubation, live alginate-printed cells were stained with calcein AM (1 µM) and cell fluorescence was acquired with the Cell Voyager CV8000 (Yokogawa). A z-stack imaging with a 4X objective lens was performed to capture 50–60 images in 20-µm steps. The maximum intensity projection (MIP) images were recorded for image analysis. MBD cell analyzer software was used for quantitative analysis of acquired images. The total area of the MIP images of drug-treated cells normalized to DMSO-treated cells was used to calculate cell viability. Dose-Response Curves (DRC) and the absolute half-maximal inhibitory concentrations (IC_50_) were generated and the Area Under the DRCs (AUCs) were calculated in GraphPad Prism 10.3.1. With the exception of spheroids ID8_FT2, ID9_FT1 and ID10, which yielded results for only 41, 33 and 25 compounds, respectively, all other spheroids generated results from the 42 compounds in the library.

After incubation with SOC drugs or encorafenib-based treatments, the viability of Matrigel-printed cells was assessed using the CellTiter-Glo® 3D Cell Viability Assay (Promega #G9682). CellTiter-Glo was thawed and pre-warmed at room temperature before use. Equal volumes of CellTiter-Glo and pre-warmed cell culture media were mixed and 50 µl-volume of the mixture was distributed into 384-black well plates (Greiner #781091). The bottom of the plates was covered with clearline brightmax white film (Dulis #760245) and the pillar-lids containing the cells were transferred to the plates containing CellTiter-Glo® 3D. The cells were lysed by vigorous shaking of the plates on a microplate vortex mixer during 5 min. The plates were then incubated at room temperature for 25 min. Luminescence was recorded using BioTek Synergy MX Multi-mode micro-plate reader.

To determine whether a patient spheroid sample is sensitive or resistant to a drug, AUCs z-scores were calculated for all the spheroid samples per drug (AUC z-score = (AUC_patient of interest_-mean AUC_all patients_)/Standard deviation AUC_all patients_). As a general rule, if a dose-response relationship is observed, the lower the AUC z-score, the higher the sensitivity of the patient sample to the drug. This approach follows the methodology established in previous studies^46,72^.

### Flow cytometry

The spheroids were dissociated using trypsin digestion. The cells were resuspended in PBS, and incubated with Zombie NIR viability dye (Biolegend #423105) in U-bottom 96-well plate during 15 min at room temperature. The cells were washed and resuspended in MACS buffer (Miltenyi #130-091-221) containing Human TruStain FcX™ Fc Receptor Blocking Solution (Biolegend #422302, 5 µL per 100 µL). After a 10-min incubation at room temperature, anti-mouse/human CD44-PE (Biolegend #103024, dilution 1/20) and anti-human CD133-RB780 (BD #756382, dilution 1/50) antibodies were added for 30 min at room temperature. After 4 washing steps with MACS buffer, the cells were fixed with True-Nuclear™ 1X Fix Concentrate (True-Nuclear™ Transcription Factor Buffer Set, Biolegend #424401) during 45 min at room temperature, then centrifuged and washed with True-Nuclear™ 1X Perm Buffer for a total of four washes. Cells were resuspended in True-Nuclear™ 1X Perm Buffer containing mouse-CDX2-AF647 antibody (BD #560395, dilution 1/5) and incubated at room temperature during 30 min. The cells were washed four times with True-Nuclear™ 1X Perm Buffer, resuspended in PBS, and acquired on Spectral ID7000 flow cytometer. Data were analyzed using FlowJO v10.8.1.

All events were gated on cells by forward scatter (FSC)/ side scatter (SSC), doublets were excluded, and Zombie NIR negative cells were defined as viable cells. Fluorescence minus one (FMO) controls were used to determine positive staining for each marker.

### Statistical analysis

Pearson correlation was applied to analyze the correlation between drug screen results obtained with fresh and cryopreserved spheroids.

To characterize statistical differences between the effects of drug families, avoiding false replication, AUC values were averaged for drugs per patient and per family, and the mean AUC values were compared between the families using two-way analysis of variance (ANOVA) and Tukey post-hoc (model: *mean_AUC* = *µ* *+* *Patient* *+* *DrugFamily* + *ɛ*). Three-way ANOVA and Tukey post-hoc were used to compare the anti-EGFR drug screen results in the clinically resistant and responsive patients’ groups (model: *AUC* = *µ* *+* *Group* *+* *Drug* *+* Group*Drug *+* *Patient* + *ɛ*). Normality of the AUC values was assessed using Shapiro test (after correction for effects of drugs and groups). Potential interactions between AUCs and percentage of CD44+, CD133+ and CD44+/CD133+ cells were addressed using Pearson correlation analysis and ANCOVA-style mixed model (*AUC* = *µ* *+* *Drug* *+* *Patient* + *β∙Marker* + *ɛ, where Marker is percentage of corresponding cells with surface markers*). The analysis was performed in R/Bioconductor (v.4.4.2). The data analysis scripts are freely available upon request.

## Supplementary information

Supp Fig and Tab_rev

Supp Data 1- variant list.

Supp Data 2- raw TSO data.

## Data Availability

IHC results of ID3 tumor samples performed for clinical purposes before patient inclusion in this study are available from the corresponding author upon request. All the other data generated and analyzed in the frame of this study were included in the article and its supplementary information files.
