# Supplementary material for: Predicting therapeutic responses in metastatic colorectal cancer through personalized functional profiling of patient-derived spheroids
Source: NPJ Precis Oncol. 2026 Mar 19;10:181. doi: 10.1038/s41698-026-01356-7 (PMC13168235; doi:10.1038/s41698-026-01356-7)
Supplement: Supplementary file 1 — Supp Fig and Tab_rev [file 41698_2026_1356_MOESM1_ESM.pdf]

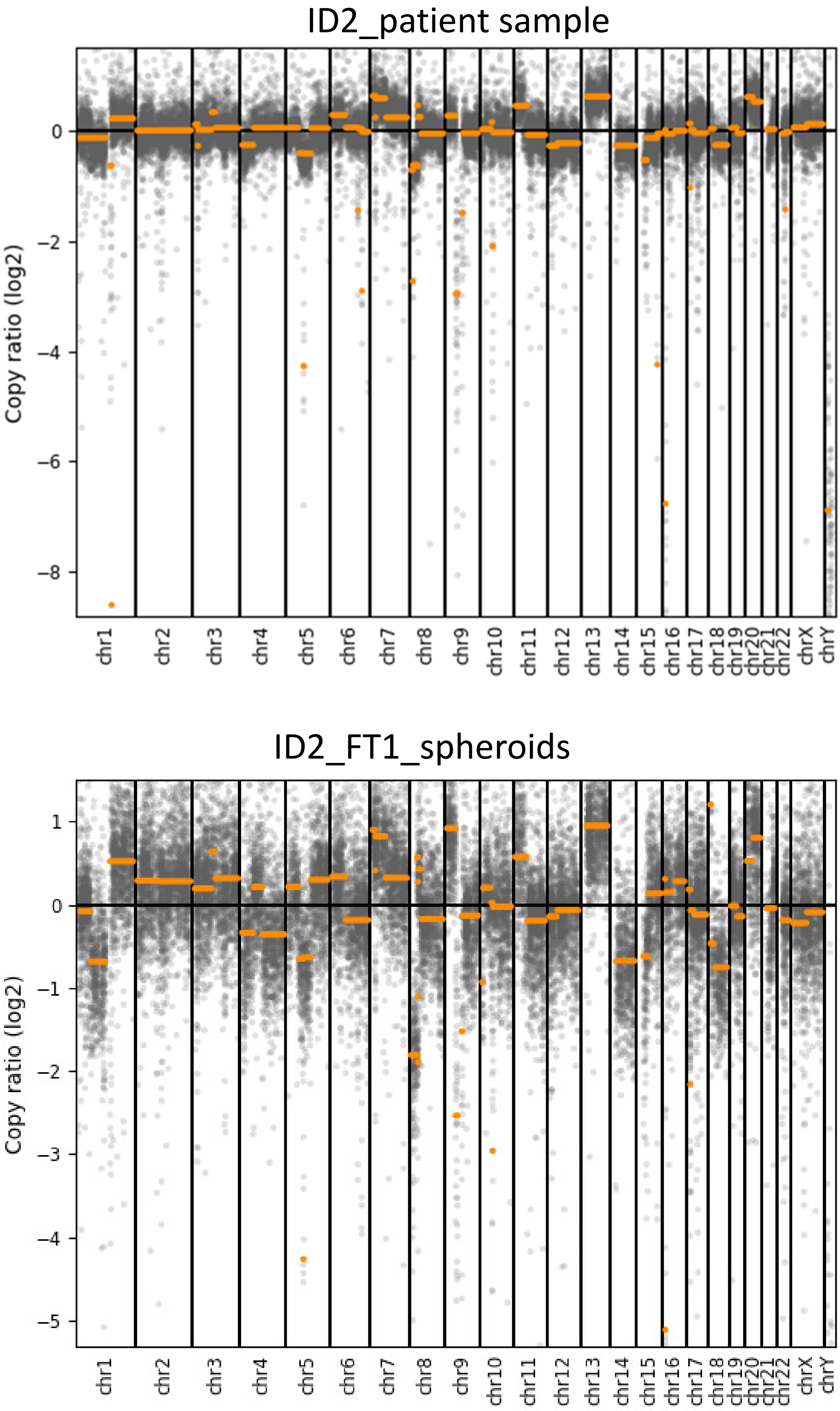

Supplementary Figure 1. Comparative Copy Number Variation (CNV) analysis between patient tumors and corresponding spheroid samples. The CNV profiles of primary tumor samples and their matched spheroids reveal a high degree of similarity. Across the cohort, CNV patterns observed in the primary tumors are mirrored in the spheroid counterparts, supporting the robustness of the spheroid model for studying copy number alterations in patient-specific contexts. CNV patterns of ID2, ID3, ID4, ID8 and ID9 patient samples and corresponding spheroids are shown.

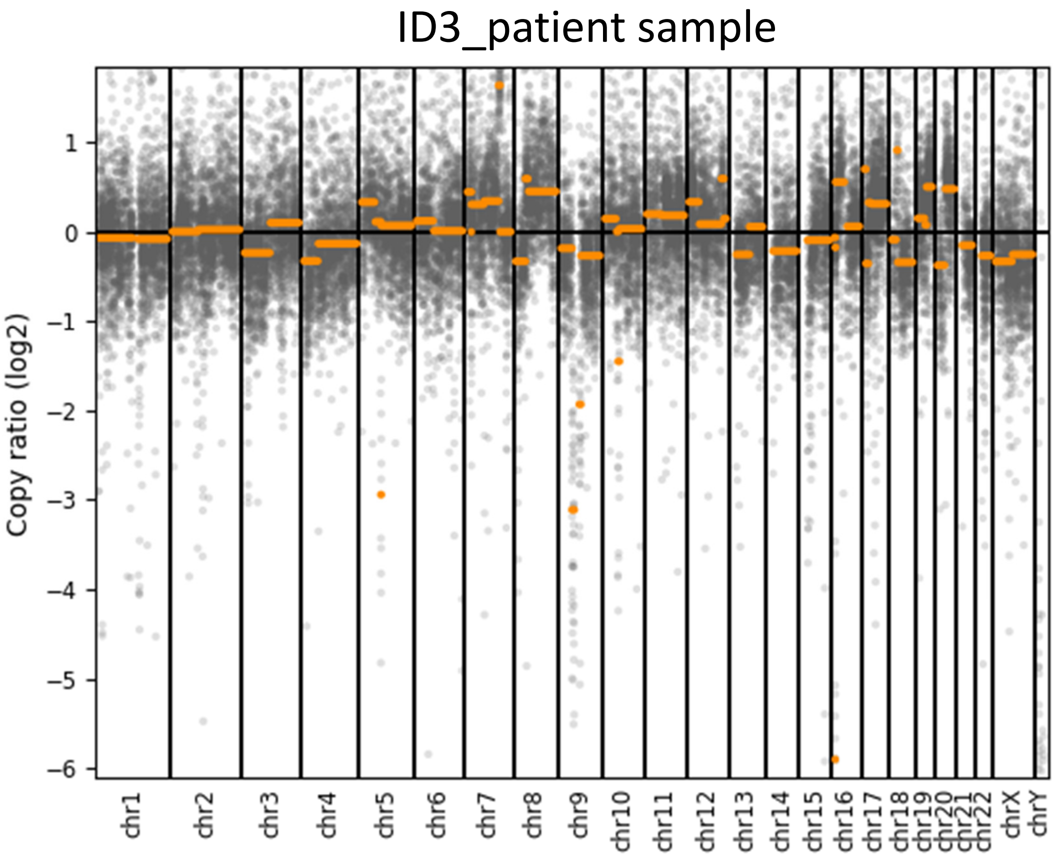

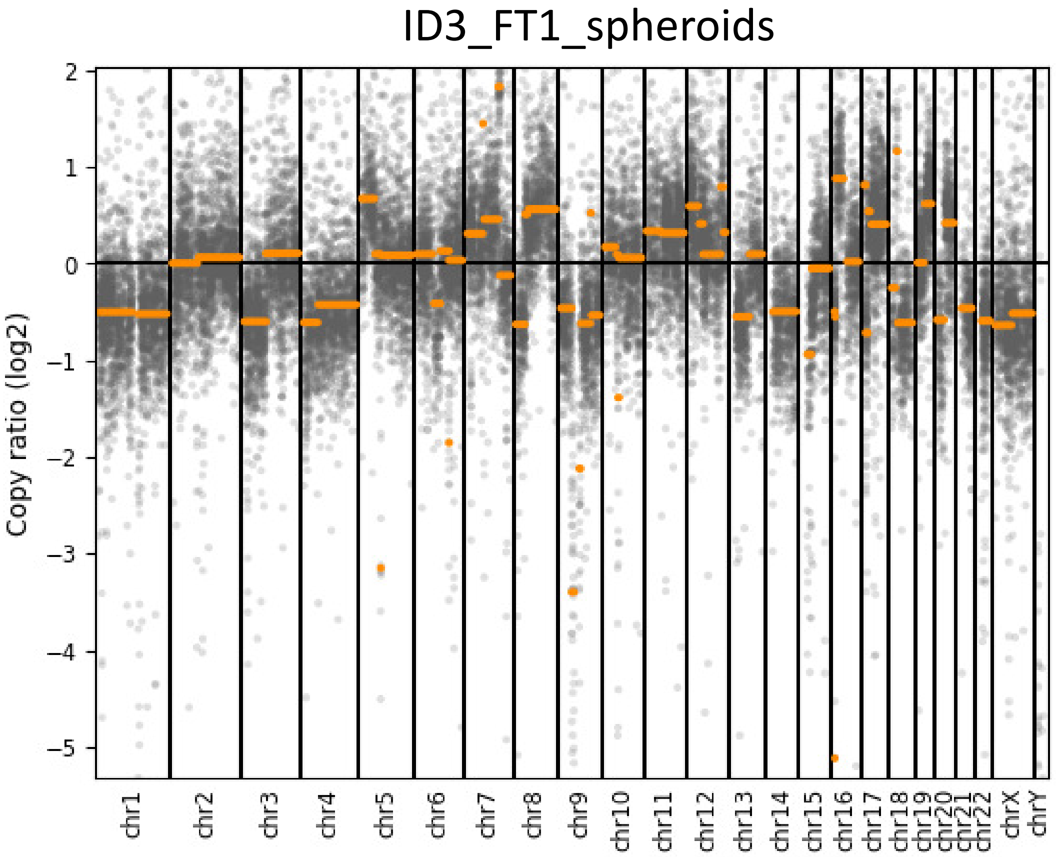

Supplementary Figure 1. *continued*

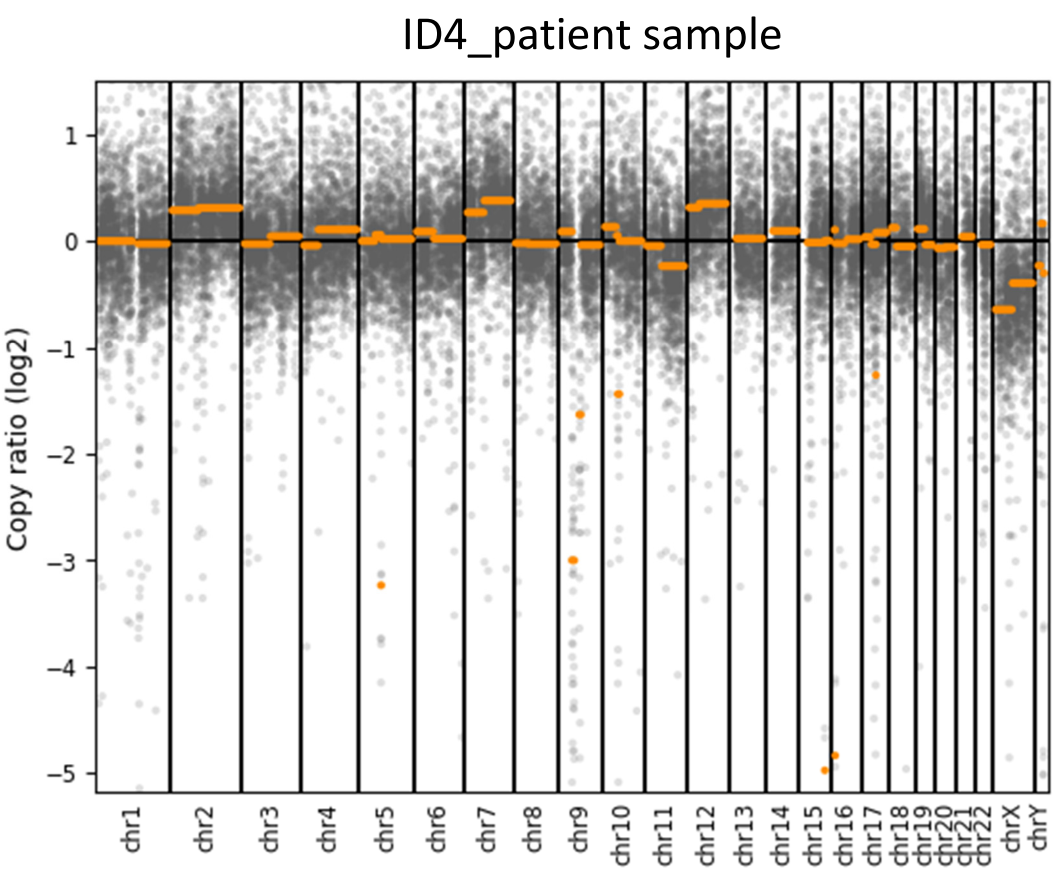

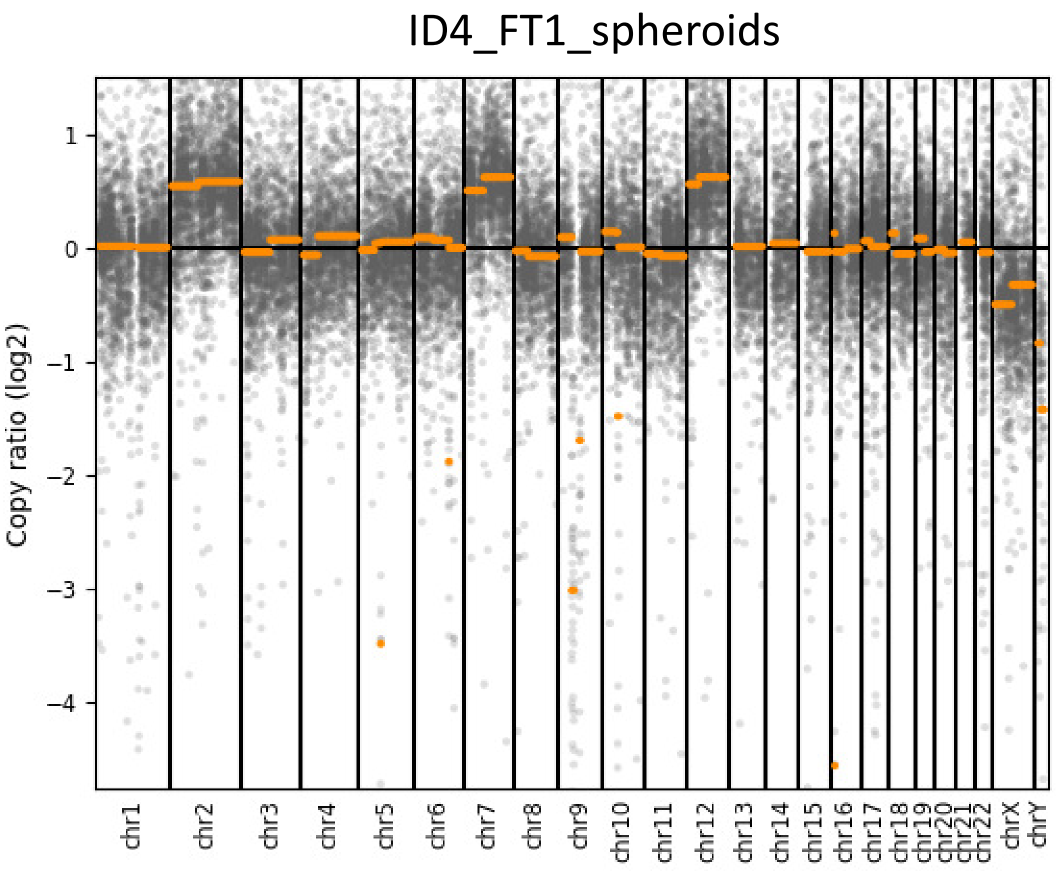

Supplementary Figure 1. *continued*

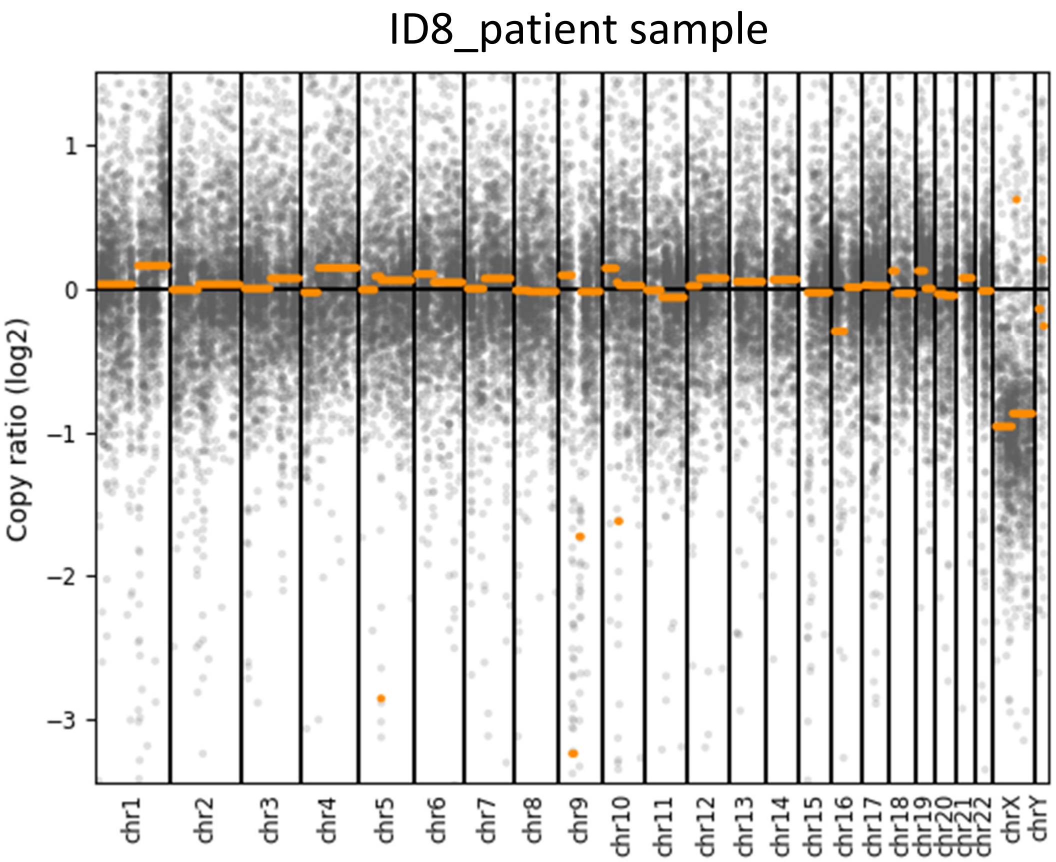

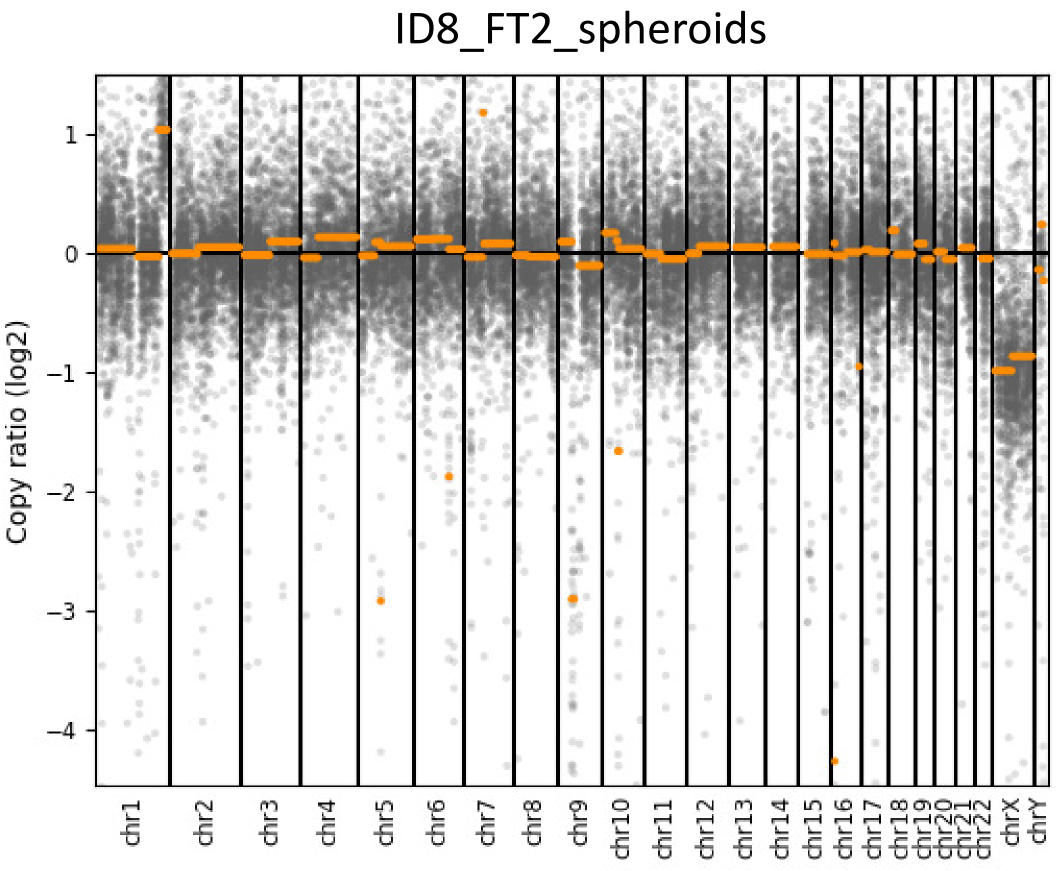

Supplementary Figure 1. *continued*

ID9_patient sample

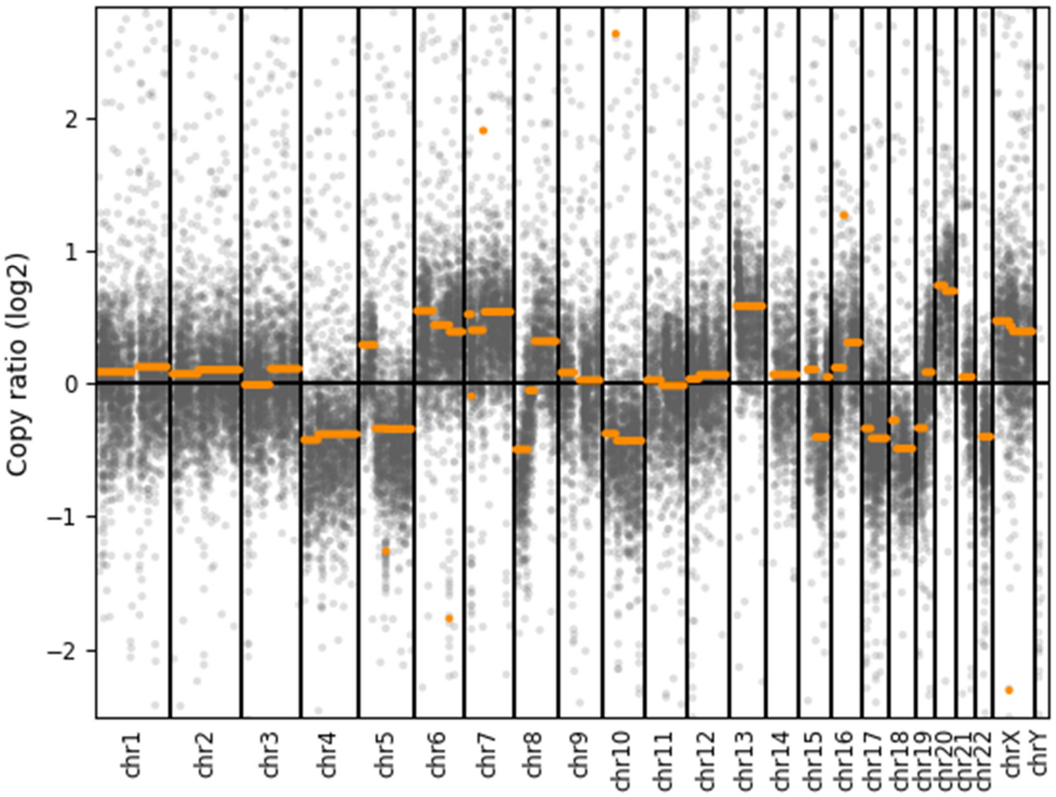

ID9_FT1_spheroids

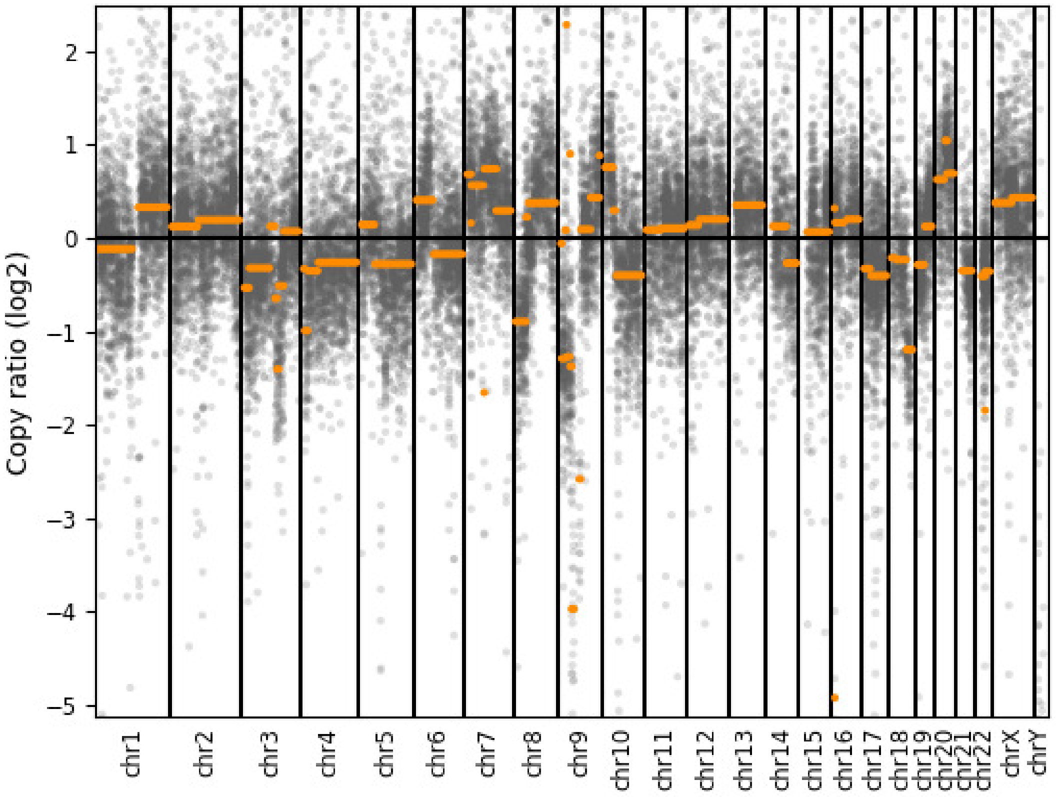

Supplementary Figure 1. *continued*

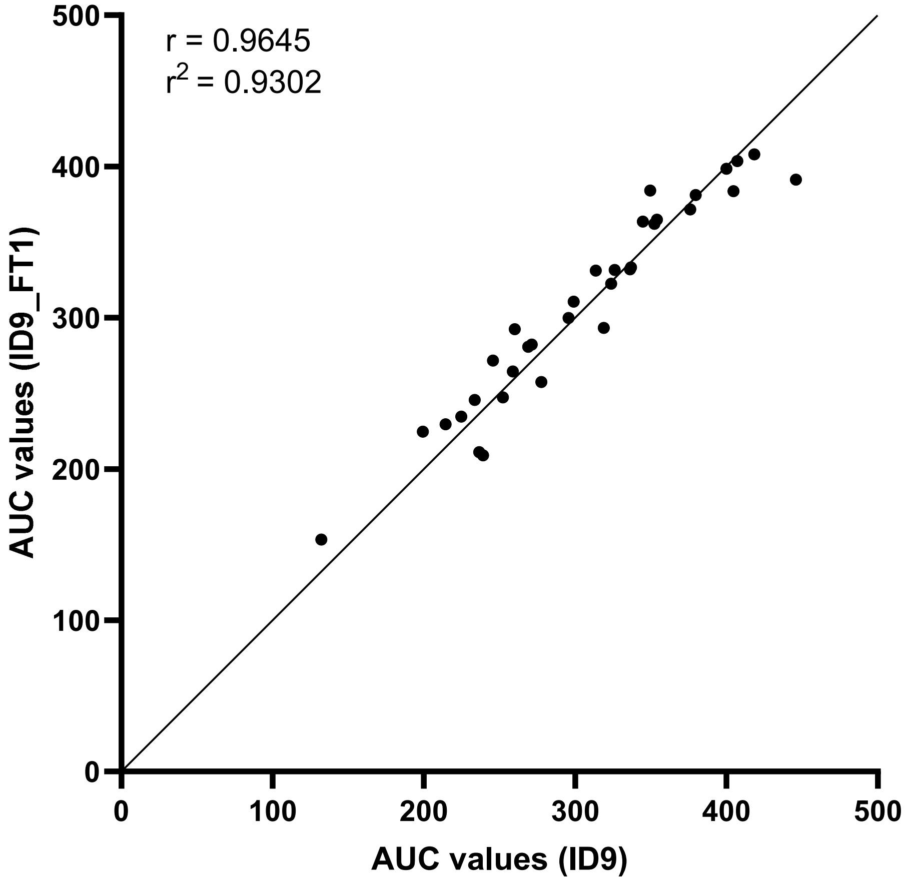

Supplementary Figure 2. Correlation of drug screen AUC values obtained with fresh and cryopreserved ID9 spheroids. Fresh ID9 and cryopreserved ID9_FT1 spheroids were subjected to a 5-day incubation with 33 compounds from the 42-drug library. The scatter plot displays the AUC values derived from the respective dose-response curves. Each dot corresponds to AUC values obtained with one compound in each of ID9 and ID9_FT1 spheroids. The line of identity is shown. Pearson correlation coefficient r and r^2 are indicated.

a

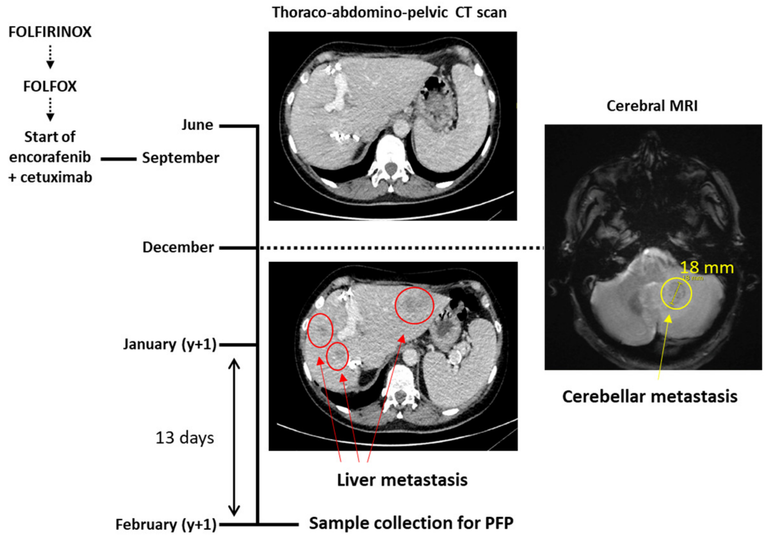

b

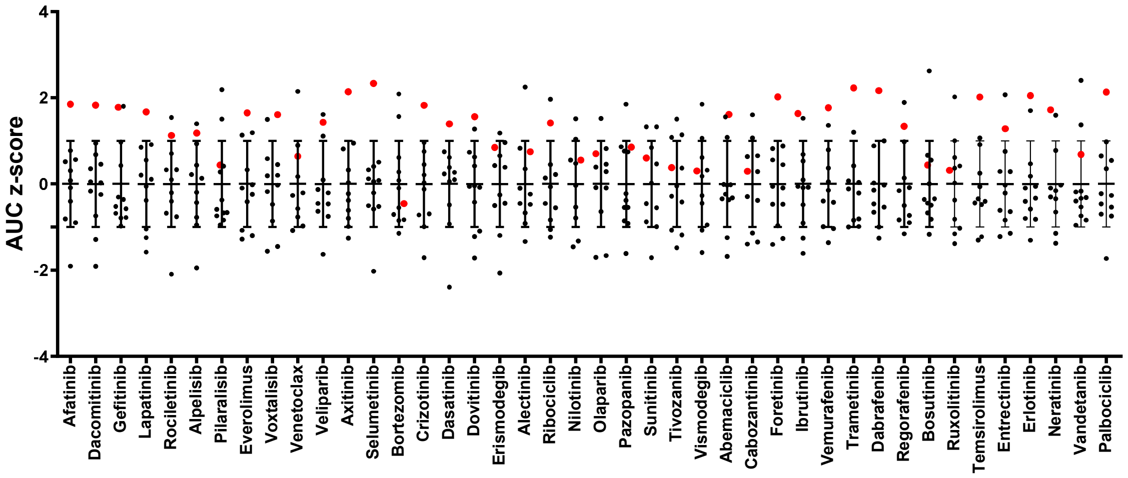

Supplementary Figure 3. Personalized drug screen results reflect the resistance of ID3_FT1 spheroids to BRAF inhibitors, in accordance with clinical response to the combination of encorafenib and cetuximab. a) Patient ID3 received encorafenib in combination with cetuximab after being treated with classical chemotherapy. During the course of the targeted therapy, a cerebral MRI and a thoraco-abdomino-pelvic CT scan were performed. (Left panel) A CT scan showing new liver metastases (bottom panel) compared to a CT scan performed 5 months earlier (upper panel). (Right panel) A cerebral MRI showing a cerebellar metastasis of 18 mm-diameter. A metastatic lymph node sample was collected for the study 13 days after the results of the last CT scan. b) mCRC patient-derived spheroids (N = 12 patients) were incubated with the 42-drug library. The scatter plot shows normalized AUCs (z-scores from the 12-patient dataset) for the 42 indicated drugs. Each dot represents a patient sample. The red dot correspond to ID3_FT1. c) Dose-response curves displaying the resistance of ID3_FT1 spheroids to the BRAF inhibitors and to regorafenib targeting among others BRAF p.V600E. The mCRC spheroids were incubated with the drugs at 7 concentrations for 5 days. Cell viability was measured using calcein AM assay and the area of the maximum intensity projection image was recorded. For each drug concentration, the cell viability was normalized to the viability of the vehicle-treated control cells. d) mCRC patient-derived spheroids (N = 8 patients) were incubated with encorafenib, encorafenib+cetuximab, encorafenib+gefitinib and encorafenib+erlotinib at 7 concentrations and fixed ratios for 3 days in matrigel. Cell viability was measured using the CellTiter-Glo® 3D Cell Viability Assay. For each treatment condition, the cell viability was normalized to the viability of the vehicle-treated control cells. Dose-response curves are shown for the 4 treatment conditions and the 8 patient-derived spheroids. ID3_FT1 results are shown in red.

c

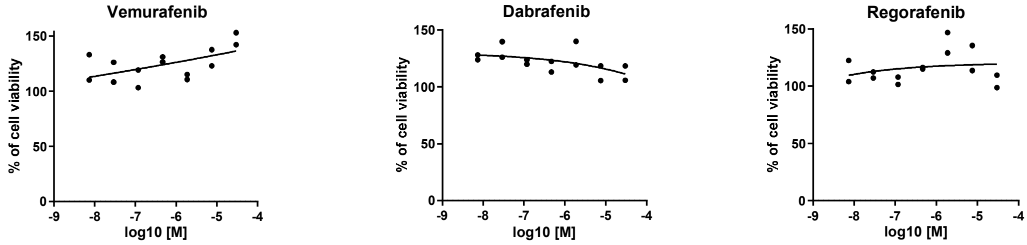

d

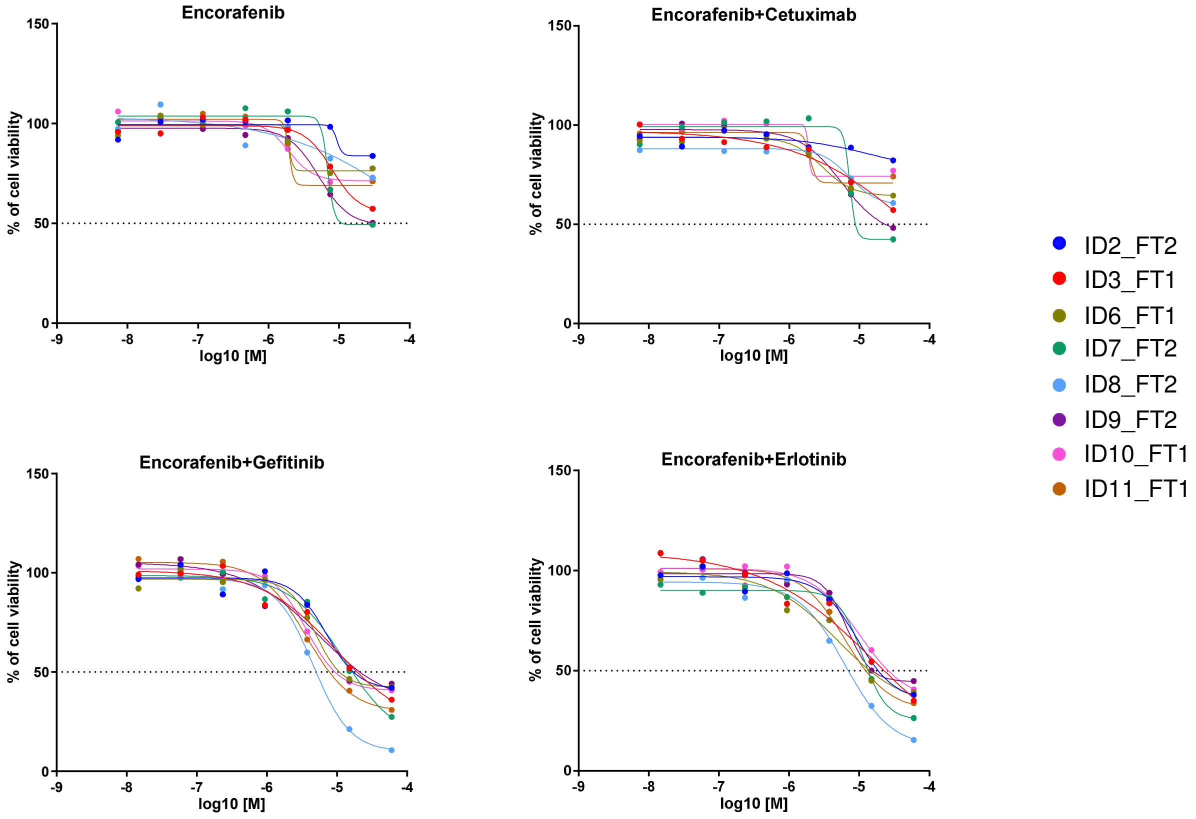

Supplementary Figure 3. *continued*

a

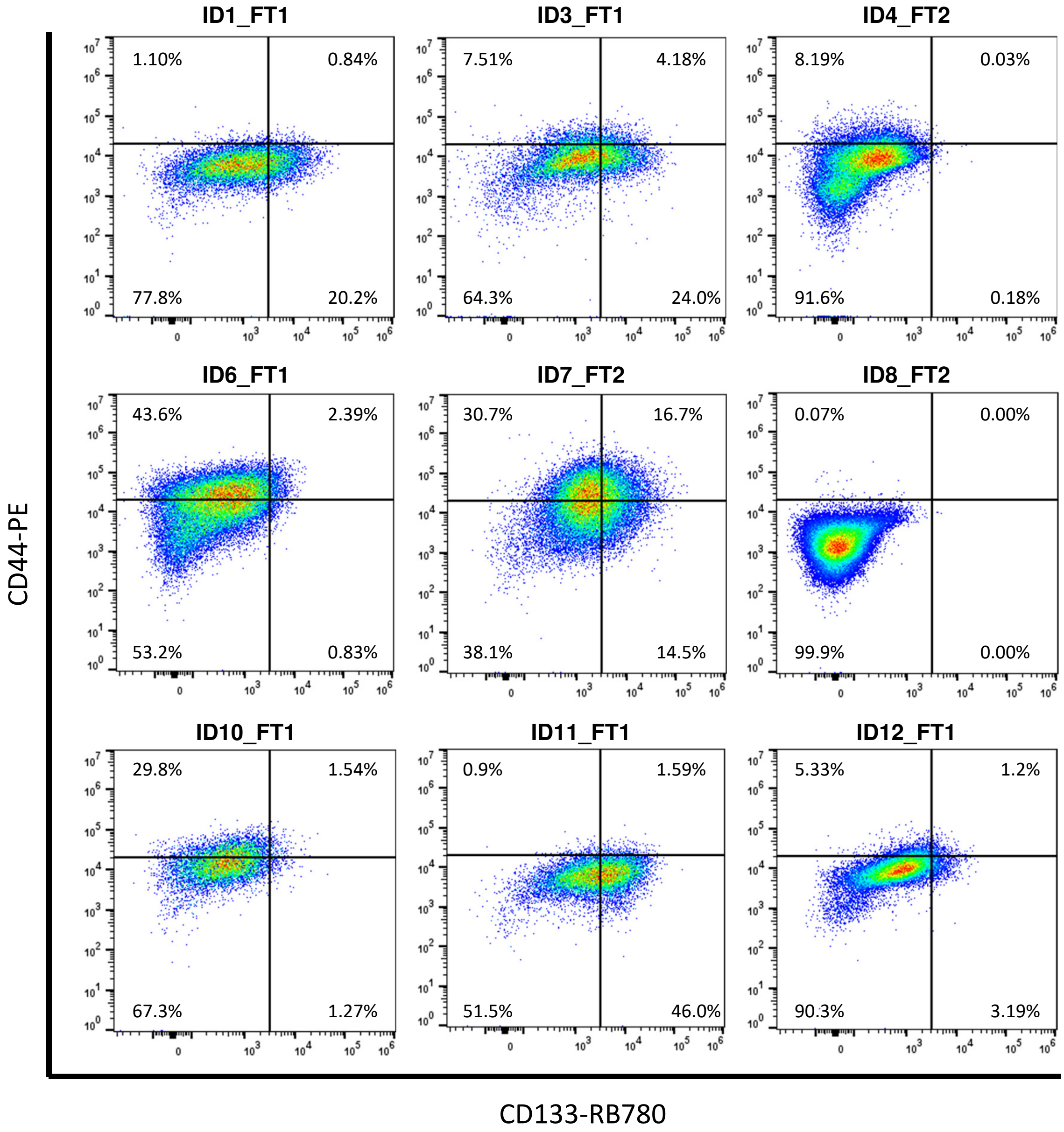

Supplementary Figure 4. Expression profiles of CD44, CD133 and CDX2 in mCRC patient-derived spheroids, as assessed by flow cytometry. Nine patient-derived spheroid samples were dissociated and labeled with RB780-conjugated CD133, PE-conjugated CD44 and AF647-conjugated CDX2. The staining results were analyzed with FlowJo and Fluorescence Minus One (FMO) controls were used to set positive cell gating. Flow cytometry plots of CD44+/CD133-, CD44-/CD133+, CD44+/CD133+ and CD44-/CD133- populations (a) and CDX2 staining (b) are shown. Percentages are indicated for each cell population.

b

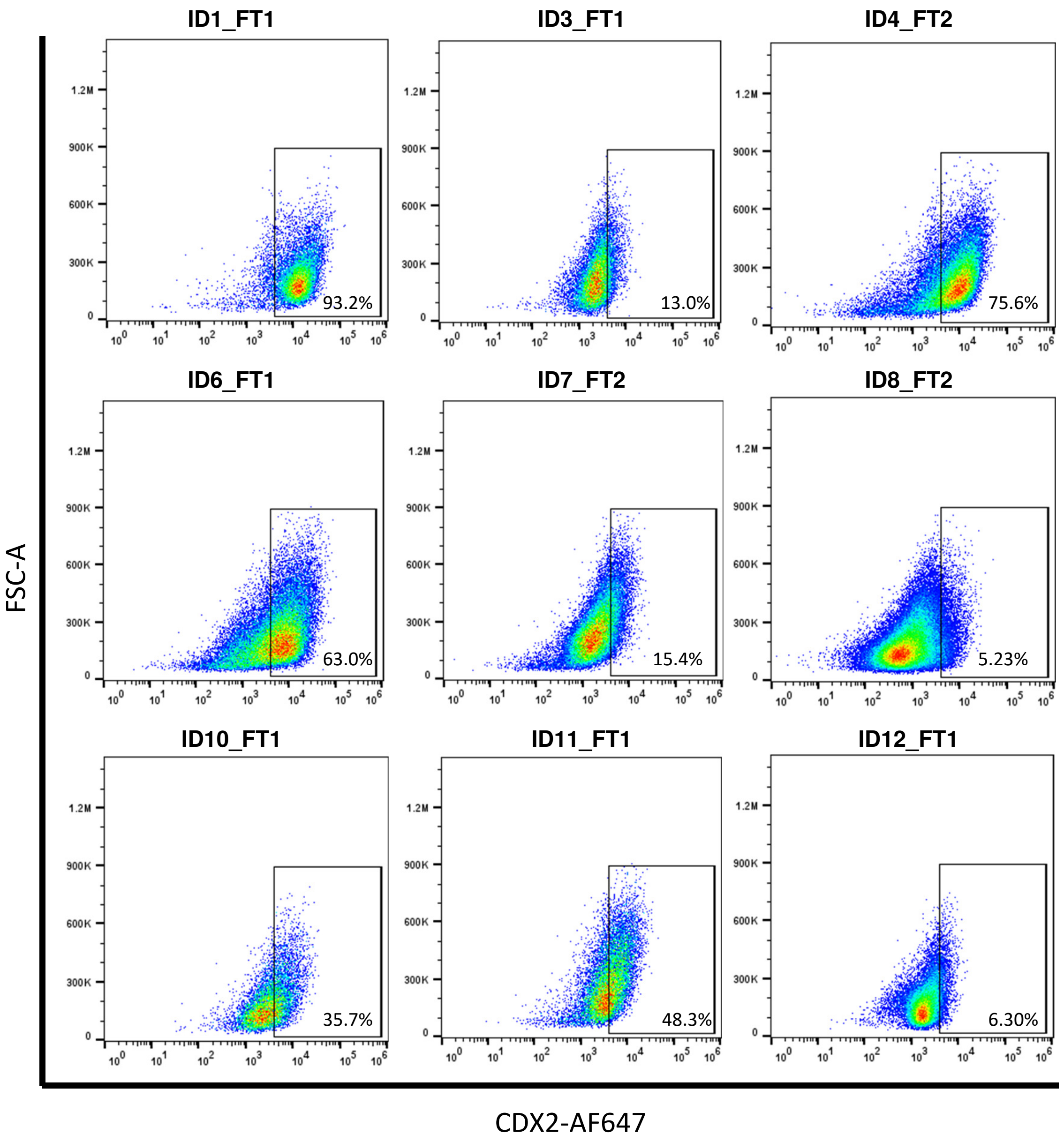

Supplementary Figure 4. *continued*

Supplementary Figure 5. Correlation of drug screen AUC values obtained with the 42-drug library and the SOC drugs, and the percentages of CD44+, CD133+ and CD44+/CD133+ cells in the spheroids. The scatter plots correspond to the analysis of the correlation between the percentages of CD44+, CD133+ and CD44+/CD133+ cells in patient-derived spheroids and the AUC values obtained with the 42-drug library and the SOC molecules using the same patient-derived spheroids. Pearson correlation coefficients (r) and p values and indicated.

Pearson correlation analysis: AUC (42-drug library) vs CD44+ (%)

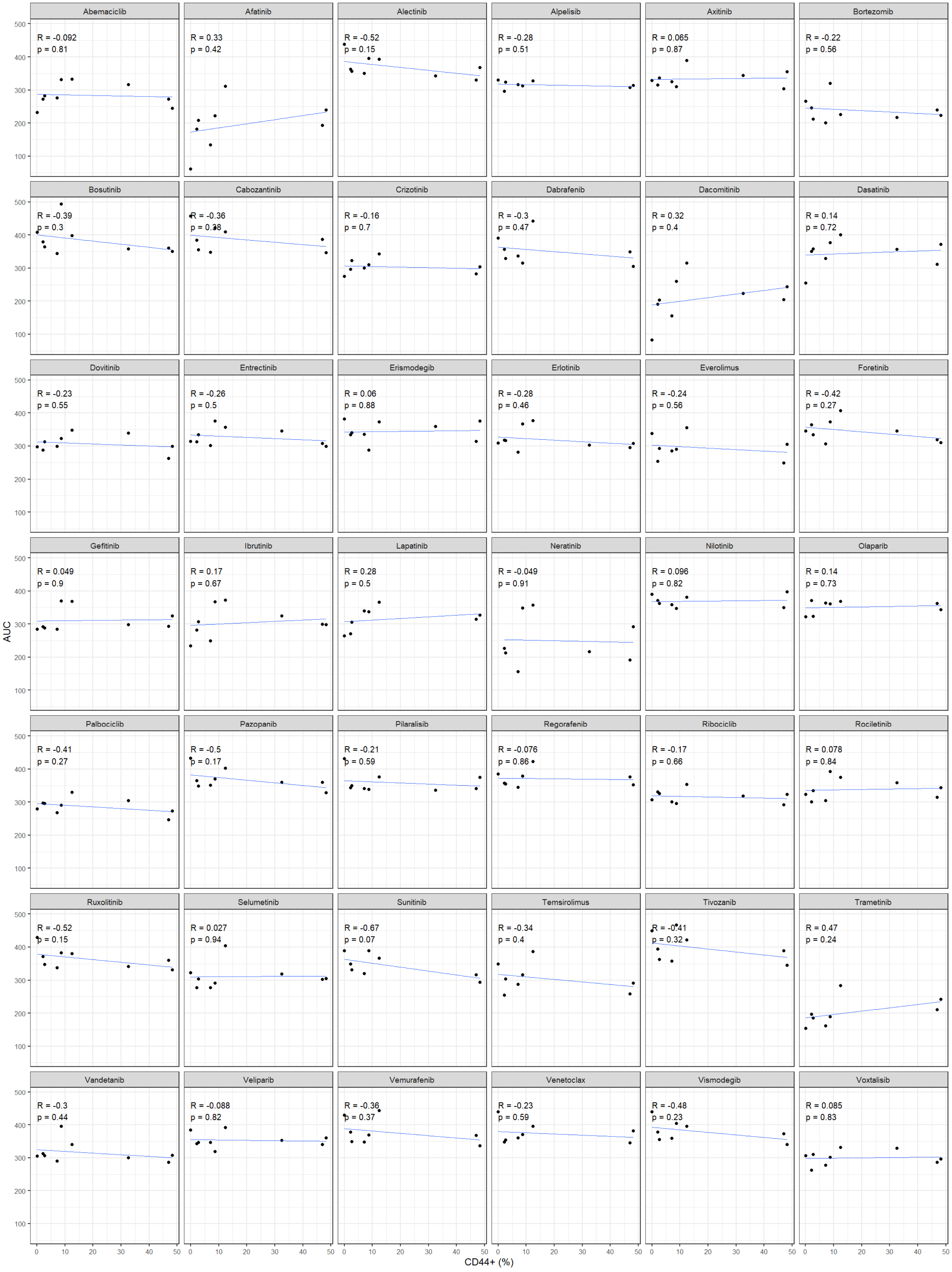

Pearson correlation analysis: AUC (42-drug library) vs CD133+ (%)

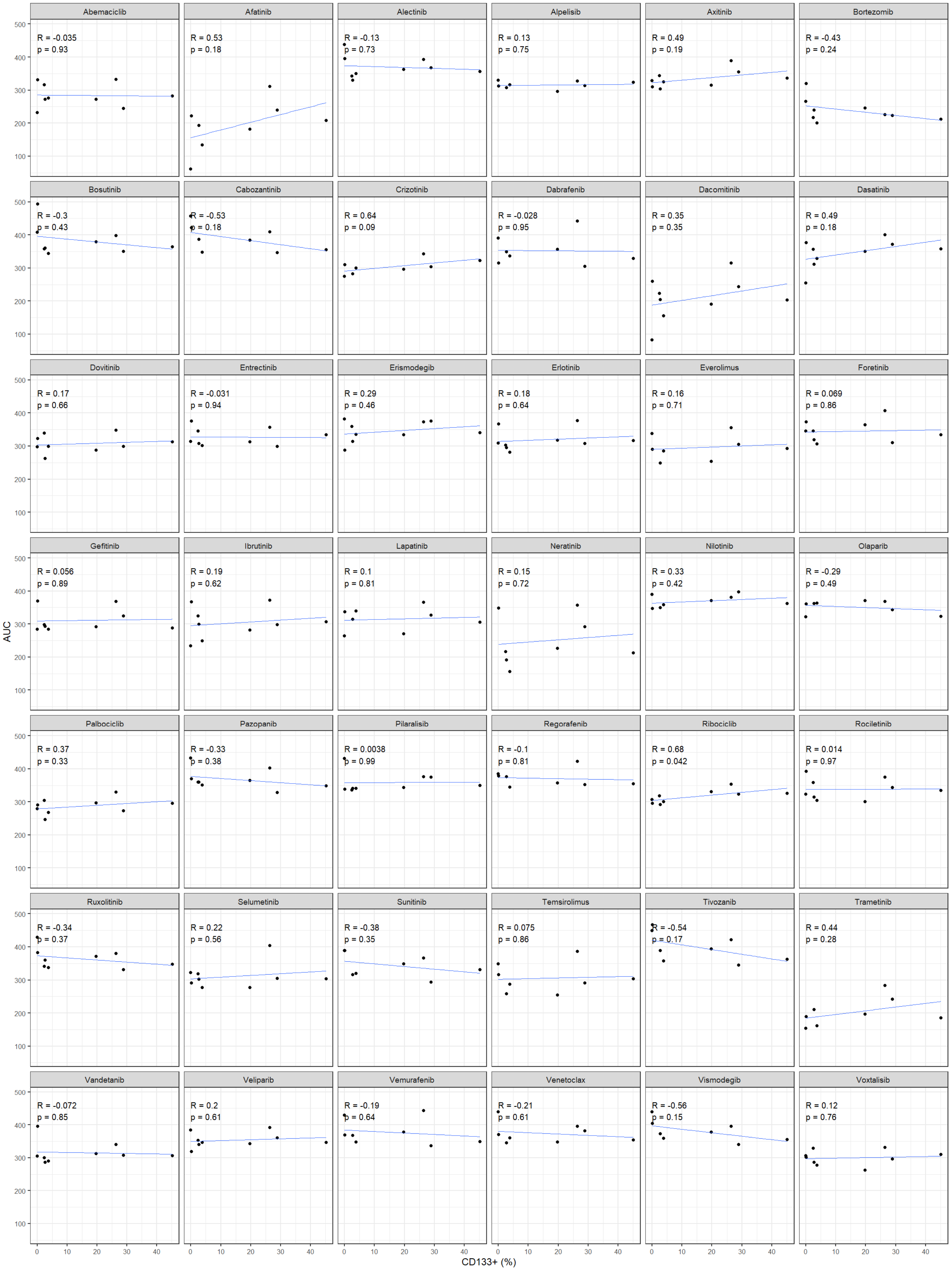

Supplementary Figure 5. *continued*

Pearson correlation analysis: AUC (42-drug library) vs CD44+/CD133+ (%)

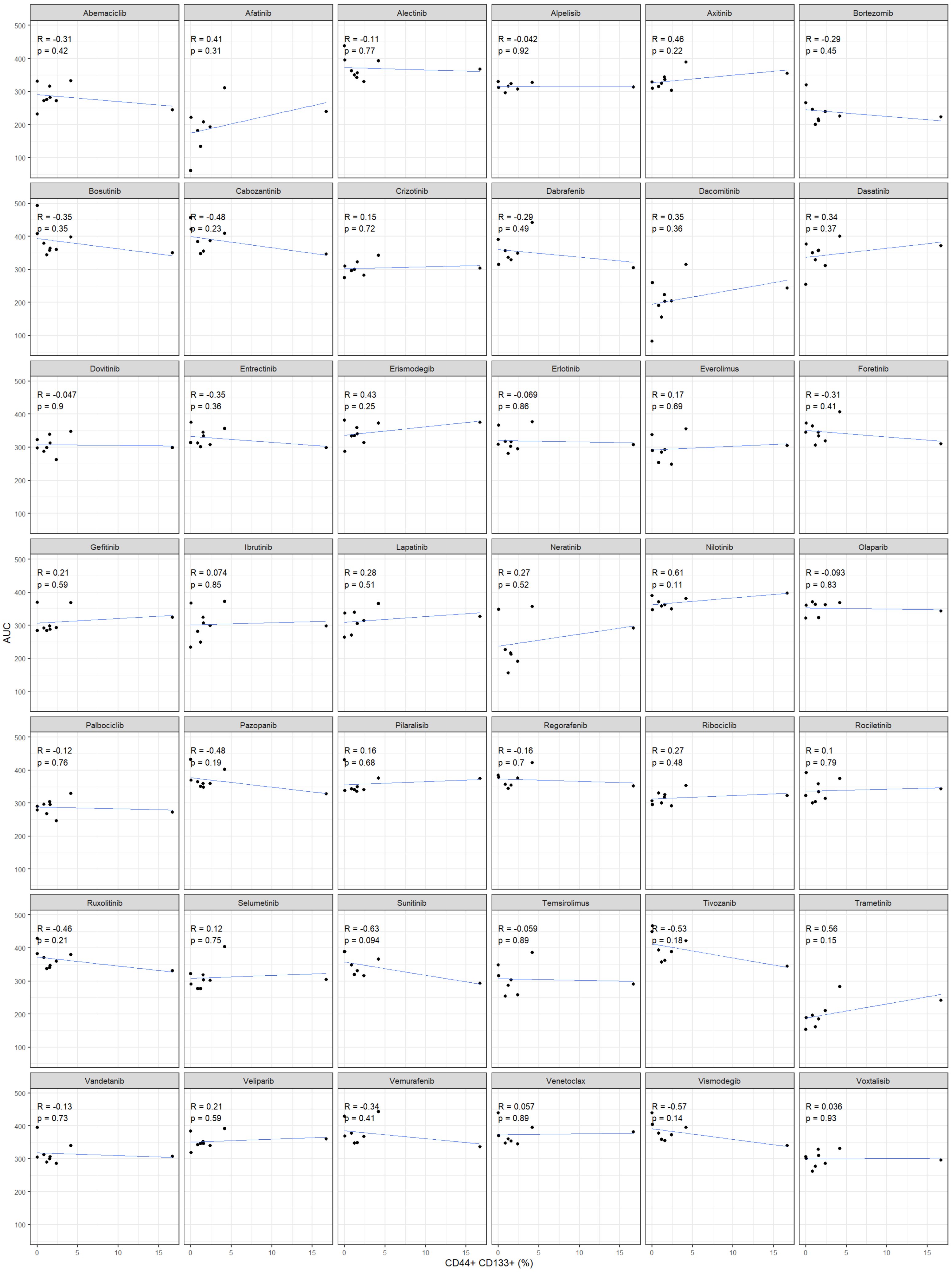

Supplementary Figure 5. *continued*

Pearson correlation analysis: AUC (SOC-drug library) vs CD44+ (%)

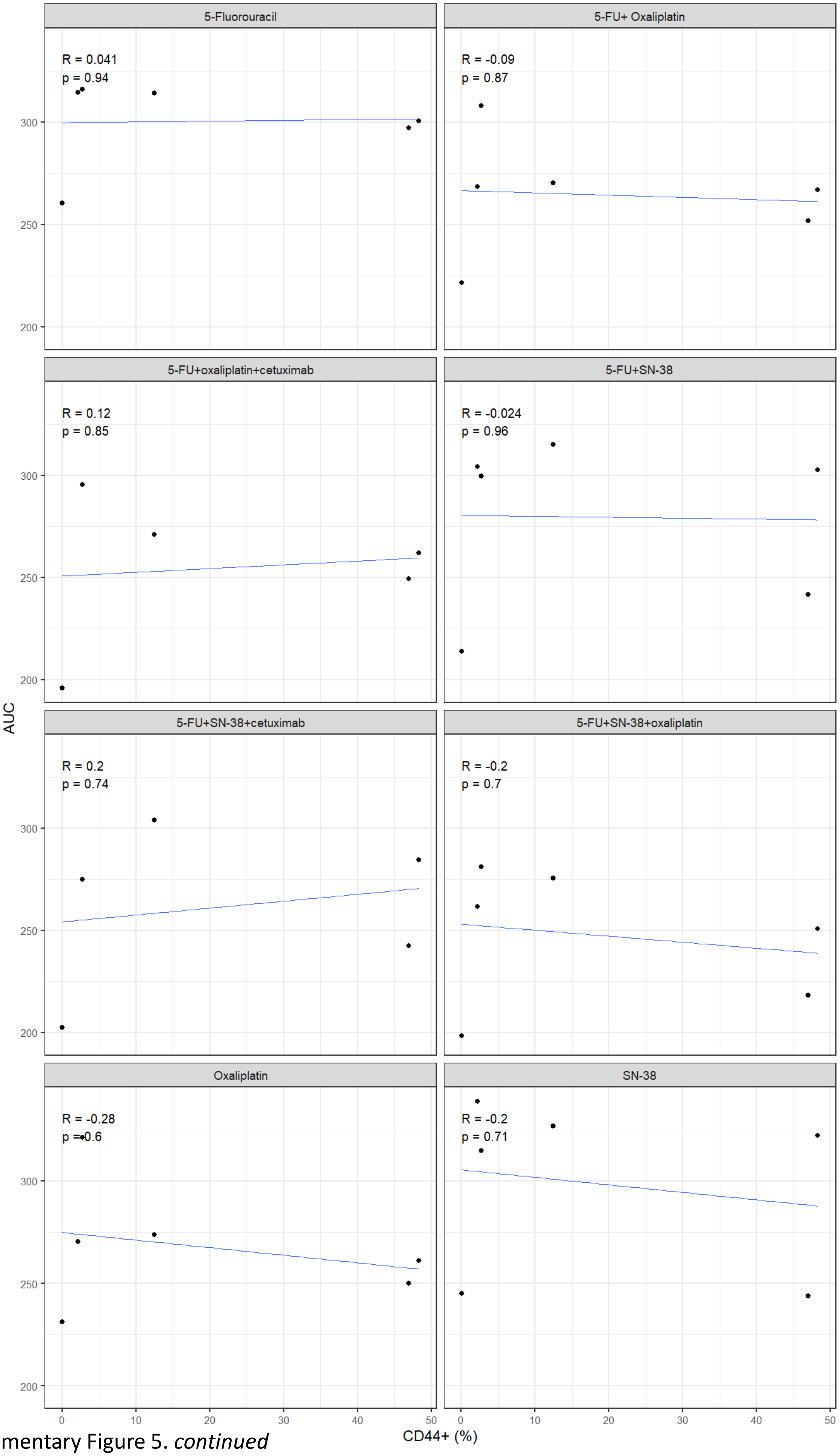

Supplementary Figure 5. *continued*

Pearson correlation analysis: AUC (SOC-drug library) vs CD133+ (%)

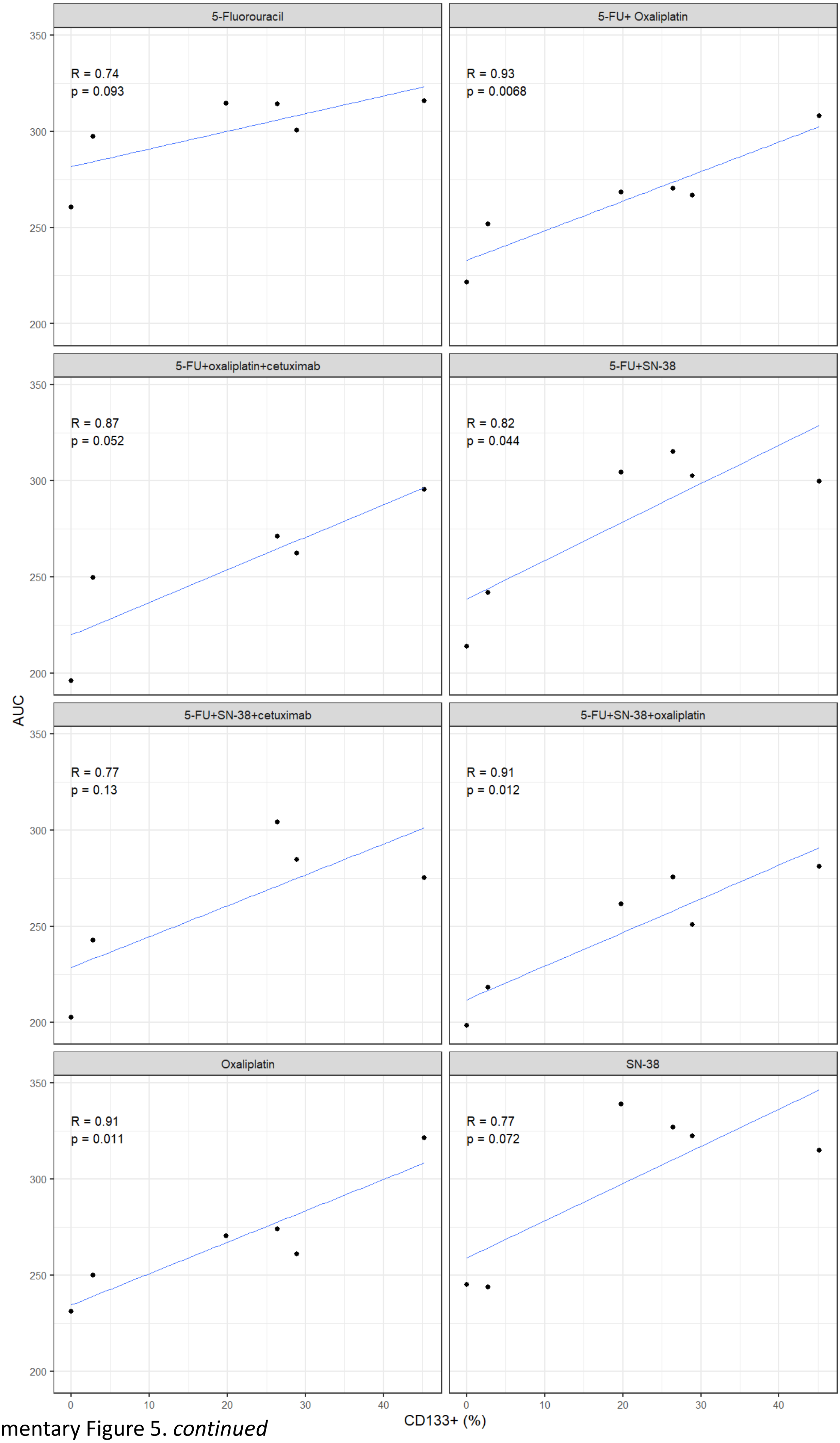

Supplementary Figure 5. *continued*

Pearson correlation analysis: AUC (SOC-drug library) vs CD44+/CD133+ (%)

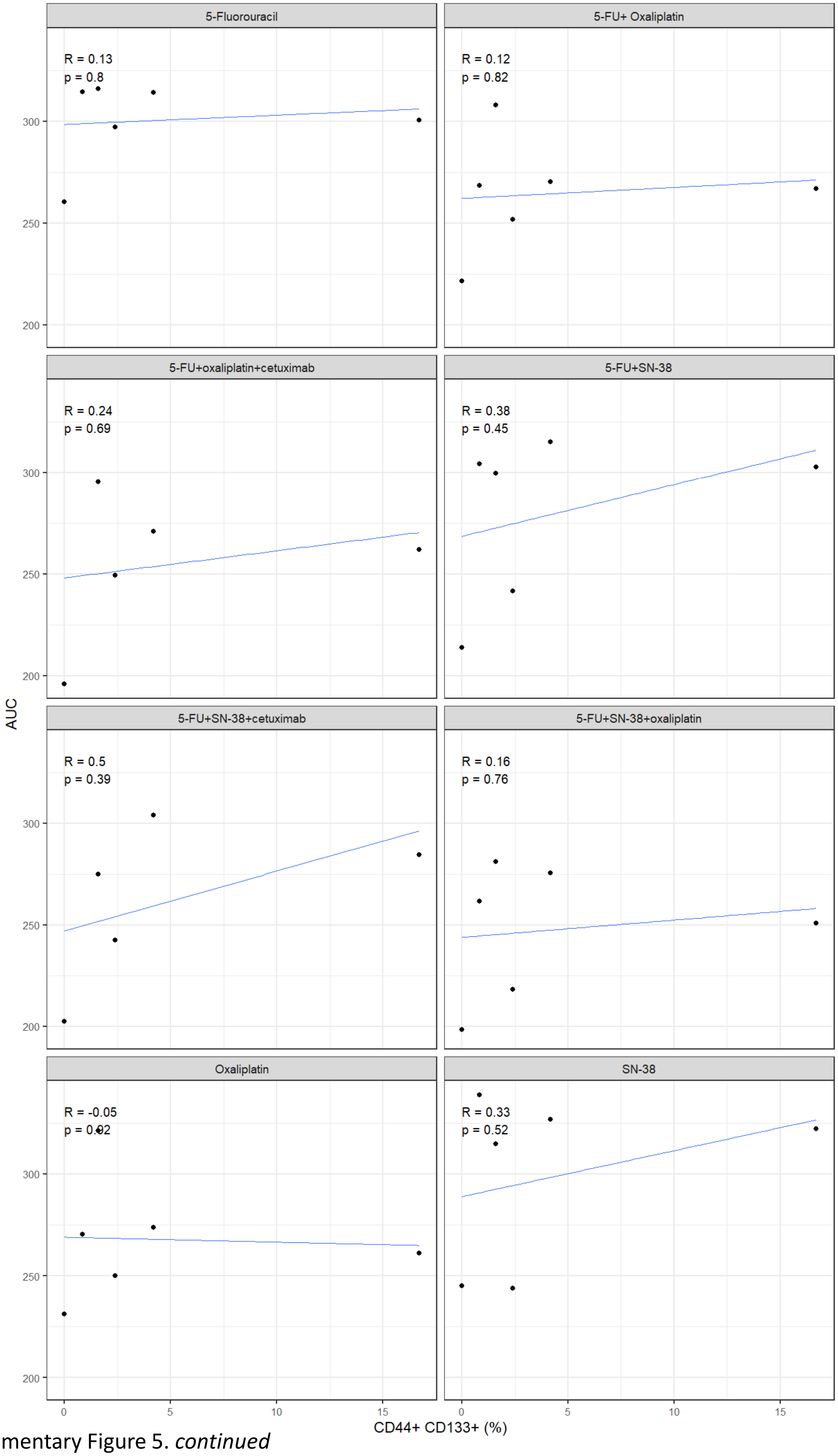

Supplementary Figure 5. *continued*

Supplementary Table 1. Descriptive metrics of the drug screening process using fresh spheroids

Patient-derived spheroid identification number	Time to cell printing (days)	Turnaround time from sample collection to drug screen results release (days)
**ID4**	29	36
**ID5**	30	37
**ID9**	29	41
**ID10**	38	70
**ID11**	106	112
**ID12**	39	83

Supplementary Table 2. Overview of shared and unique characteristics between tumors and derived spheroids.

	ID2_patient and ID2_FT1_spheroid	ID3_patient and ID3_FT1_spheroid	ID4_patient and ID4_FT1_spheroid	ID8_patient and ID8_FT2_spheroid	ID9_patient and ID9_FT1_spheroid	ID10_patient and ID10_spheroid
**Matched variants**	8	13	19	61	6	11
**Unmatched variants_patient sample**	16	-	17	68	3	3
**Unmatched variants_spheroids**	12	-	21	30	9	4
**CNVs (Overall)_ patient sample**	76	66	57	53	59	-
**CNVs (Overall)_ spheroids**	69	71	53	58	75	-
**CNAs (fold change >3)_patient sample**	-	1	-	-	-	-
**CNAs (fold change >3)_spheroids**	-	1	-	-	-	-
**TMB (mut/mb)_ patient sample**	9,4	3,9	21,2	65	0	1.6
**TMB (mut/mb)_ spheroids**	16,4	2,3	14,9	68,8	4,7	3.9
**Fusions_patient sample**	-	-	-	-	-	-
**Fusions_spheroids**	-	-	-	-	1	-
**RNA alternative transcripts_patient sample**	-	1	-	-	-	-
**RNA alternative transcripts_spheroids**	-	1	-	-	-	-
**MSI score_patient sample**	3,31	2,4	0	76,42	3,23	2.46
**MSI score_spheroids**	4,88	2,44	1,6	88	2,4	1.65

MSI score = percent unstable MSI sites

Supplementary Table 3. Drug library: compounds, corresponding supplier references and main targets

Selleckchem Supplier reference	Compound	Main targets
S7158	Abemaciclib	CDK4/6
S1011	Afatinib	EGFR, HER2
S2762	Alectinib	ALK
S2814	Alpelisib	PI3K
S1005	Axitinib	VEGFR1/2/3
S1013	Bortezomib	Proteasome
S1014	Bosutinib	BCR-ABL, SRC
S1119	Cabozantinib	VEGFR1/2/3, MET, RET, KIT, FLT3, AXL
S1068	Crizotinib	MET, ALK, ROS1
S2807	Dabrafenib	BRAF(V600E/K/D)
S2727	Dacomitinib	EGFR, HER2
S1021	Dasatinib	BCR-ABL, SRC
S1018	Dovitinib	FGFR1/3, VEGFR1/2/3, PDGFRβ, FLT3, KIT
S7998	Entrectinib	TrkA/B/C, ROS1, ALK
S2151	Erismodegib	Hedgehog/smoothened
S1023	Erlotinib	EGFR
S1120	Everolimus	mTOR
S1111	Foretinib	MET, VEGFR
S1025	Gefitinib	EGFR
S2680	Ibrutinib	BTK
S2111	Lapatinib	EGFR, HER2
S2150	Neratinib	EGFR, HER2
S1033	Nilotinib	BCR-ABL, KIT, PDGFR, DDR1
S1060	Olaparib	PARP1/2
S1116	Palbociclib	CDK4/6
S1035	Pazopanib	KIT, VEGFR1/2/3, PDGFR, FGFR1/3
S7645	Pilaralisib	PI3K
S1178	Regorafenib	VEGFR1/2/3, PDGFR, FGFR, KIT, RET, RAF-1, BRAF and BRAFV600E
S7440	Ribociclib	CDK4/6
S7284	Rociletinib	EGFR
S1378	Ruxolitinib	JAK1/2
S1008	Selumetinib	MEK1/2
S1042	Sunitinib	KIT, VEGFR, PDGFR
S1044	Temsirolimus	mTOR
S1207	Tivozanib	VEGFR
S2673	Trametinib	MEK1/2
S1046	Vandetanib	VEGFR2, EGFR, RET
S1004	Veliparib	PARP1/2
S1267	Vemurafenib	BRAF(V600E)
S8048	Venetoclax	BCL-2
S1082	Vismodegib	Hedgehog/smoothened
S1523	Voxtalisib	Dual PI3K/mTOR

Supplementary Table 4. Percentages of different cell populations resulting from CD44, CD133 and CDX2 flow cytometry analysis in patient-derived spheroids.

	ID1_FT1	ID4_FT2	ID6_FT1	ID7_FT2	ID8_FT2	ID10_FT1	ID11_FT1	ID12_FT1	ID3_FT1
**CD44+**	2,19	8,75	47,00	48,30	0,07	32,60	2,75	7,18	12,50
**CD133+**	19,8	0,16	2,75	28,90	0,00	2,44	45,20	3,81	26,40
**CD44+/CD133+**	0,84	0,03	2,39	16,70	0,00	1,54	1,59	1,20	4,18
**CD44+/CD133-**	1,10	8,19	43,60	30,70	0,07	29,80	0,90	5,33	7,51
**CD44-/CD133+**	20,20	0,18	0,83	14,50	0,00	1,27	46,00	3,19	24,00
**CD44-/CD133-**	77,80	91,60	53,20	38,10	99,90	67,30	51,50	90,30	64,30
**CDX2+**	93,20	75,60	63,00	15,40	5,23	35,70	48,30	6,30	13,00
**CDX2-/CD44+**	0,06	1,92	12,20	36,00	0,04	15,50	0,35	3,20	7,79
**CDX2+/CD44+**	1,79	6,10	33,30	11,10	0,02	15,30	2,07	3,09	3,66
**CDX2+/CD44-**	91,30	69,10	29,20	3,73	5,03	19,30	45,10	2,81	8,47
**CDX2-/CD44-**	6,85	22,90	25,30	49,20	94,90	49,90	52,40	90,90	80,10
**CDX2-/CD133+**	2,47	0,06	0,50	22,00	0,00	1,01	16,70	3,03	23,10
**CDX2+/CD133+**	18,30	0,15	2,63	8,69	0,00	1,64	30,40	1,22	4,62
**CDX2-/CD133-**	4,41	24,70	36,90	63,10	94,90	64,20	36,00	91,00	64,60
**CDX2+/CD133-**	74,80	75,10	59,90	6,18	5,09	33,10	16,90	4,73	7,61

Supplementary Data 1. Variant list. The genomic analysis comparing original tumor samples with their corresponding spheroids revealed a high degree of genetic fidelity. A detailed list of shared and subclonal variants, including allele frequencies and sequencing depth, is provided.

Supplementary Data 2. Raw TSO data. Raw output files from TSO pipeline are shown. Key driver mutations present in the original tumors were detected in the derived spheroids across all sample pairs. This congruence extended to structural variations (gene amplifications and fusions).
